# Biomimetic optimization of silicone breast implant integration: insights into wound healing and the foreign body response

**DOI:** 10.3389/fbioe.2025.1668930

**Published:** 2025-09-17

**Authors:** Kevin Dzobo, Traci A. Wilgus, Vanessa Zamora Mora, Audry Zoncsich, Roberto de Mezerville, Nonhlanhla Khumalo, Ardeshir Bayat

**Affiliations:** ^1^ Medical Research Council-SA Wound Healing Unit, Hair and Skin Research Laboratory, Division of Dermatology, Department of Medicine, Groote Schuur Hospital, University of Cape Town, Cape Town, South Africa; ^2^ Department of Pathology, The Ohio State University Wexner Medical Centre, Columbus, OH, United States; ^3^ Establishment Labs Holdings, Alajuela, Costa Rica

**Keywords:** wound healing, foreign body response, silicone implant, protein adsorption, inflammation, extracellular matrix, fibrosis

## Abstract

Breast augmentation is the most prevalent aesthetic surgical procedure worldwide. While silicone breast implants have evolved in terms of safety and biocompatibility, they inevitably trigger a foreign body response (FBR). This complex process can lead to fibrous encapsulation, capsular contracture, and other complications, often necessitating invasive revision surgeries. This review comprehensively analyzes the molecular and cellular mechanisms underlying FBR, emphasizing the crucial role of implant surface properties. We demonstrate how these properties, including topography, hydrophobicity, and charge, govern the initial protein adsorption patterns, effectively establishing a “molecular fingerprint” that dictates subsequent cellular interactions. This, in turn, orchestrates immune cell activation, notably macrophages, which exhibit plasticity in their polarization into pro-inflammatory (M1) and pro-fibrotic (M2) phenotypes. The balance between these phenotypes influences the extent of fibrosis and capsular contracture. We explored the five distinct phases of FBR: protein adsorption, acute inflammation, chronic inflammation, foreign body giant cell (FBGC) formation, and encapsulation. The impact of implant surface properties on each phase was elucidated, highlighting the dynamic interplay between macrophages, lymphocytes, and matrix. The phenomenon of “frustrated phagocytosis,” where macrophages fail to engulf the implant, leading to FBGC formation and chronic inflammation, is also examined. Finally, we explore promising strategies to modulate FBR and enhance implant biocompatibility, including biomimetic coatings, the use of decellularized matrices, and therapies aimed at disrupting specific molecular pathways involved in fibrosis. This review provides insights into the development of next-generation implants that can harmoniously integrate with the body, minimizing FBR and ensuring long-term clinical success.

## 1 Introduction

Millions of women worldwide have undergone breast augmentation, a procedure that addresses both cosmetic desires and reconstructive needs, by correcting breast volume and shape abnormalities. This makes it the most prevalent aesthetic surgical procedure globally (Major et al., 2015). Given that breast implants are designed to reside within the body for extended periods, understanding their complex interplay with the surrounding tissues is paramount. While silicone implants, first introduced in 1963, have undergone significant advancements in biocompatibility and safety (Gonzalez et al., 2016; George et al., 2006), they inevitably elicit a foreign body response (FBR). This intricate biological process can lead to complications such as capsular contracture, a condition that causes hardening and distortion of the breast, often requiring further surgery (Tebbetts, 2002). Although research has shown that implant modifications, like micro-texturing, can mitigate the FBR and reduce capsular contracture (Tebbetts, 2002), unfavorable outcomes persist. This underscores the critical need to further understand and modulate this response to improve patient outcomes.

**FIGURE 1:**
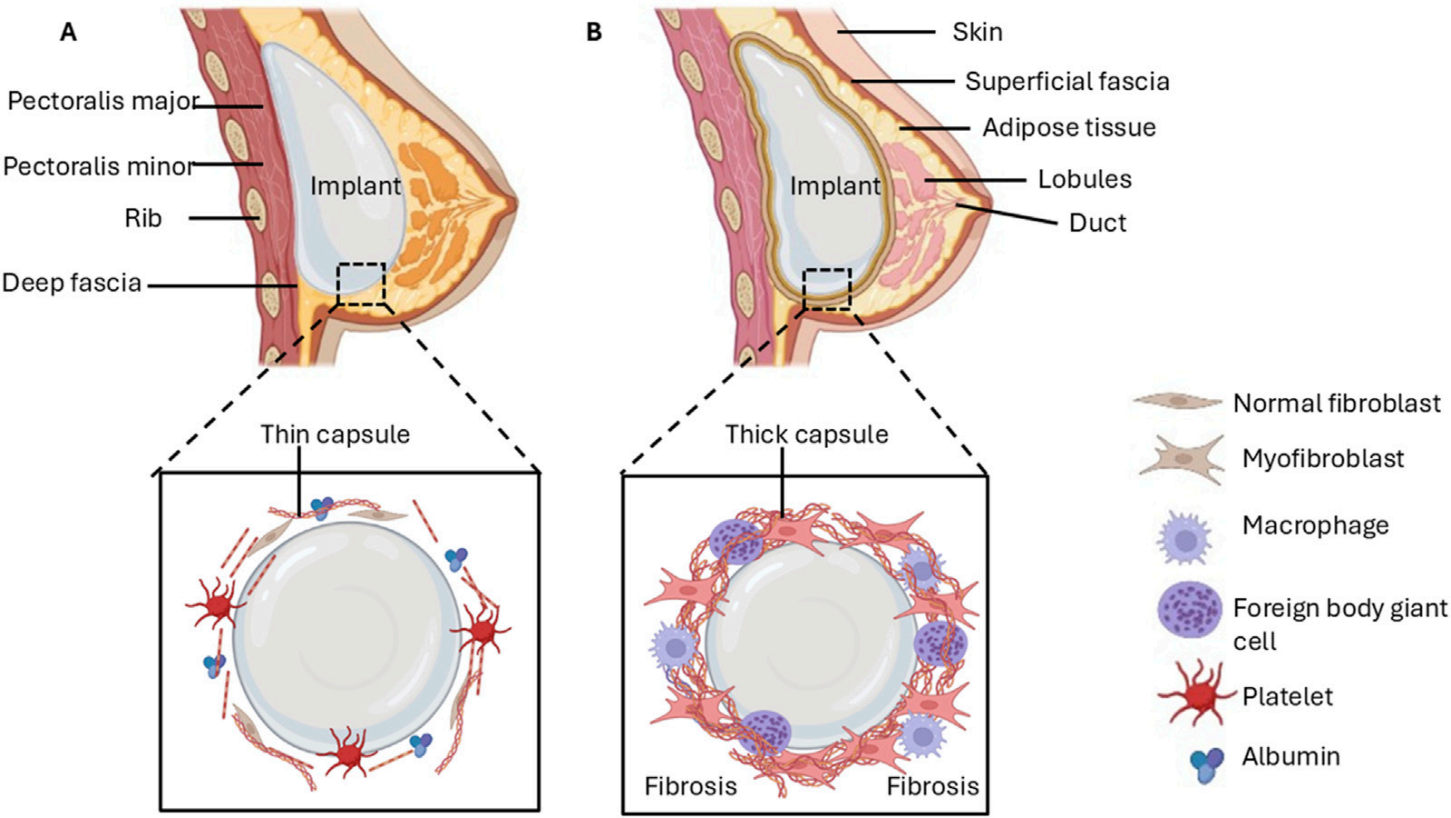
Early stages of breast implant insertion and late-stage complications due to fibrosis. **(A)** Protein adsorption on the implant surface enables the adhesion of cells, which triggers processes that form a provisional capsule around the implant. **(B)** Chronic inflammation followed by excessive synthesis of the ECM may lead to the development of a dense, contracting capsule that impedes the integration of implant and breast tissue.

The body’s reaction to a silicone implant encompasses two intertwined processes: the wound healing response triggered by the surgical trauma, and the FBR, representing the long-term interaction between the implant and the immune system (Major et al., 2015; Chandorkar et al., 2018). This review delineates both processes, dissecting their impact on potential complications, with a particular focus on fibrosis and capsular contracture formation (Figure 1). We further explore emerging strategies aimed at modulating these responses to enhance implant biocompatibility and ensure the long-term success of breast implantation, not only for aesthetic purposes but also for crucial oncological and reconstructive applications.

## 2 Methodology

Articles used in the synthesis of this manuscript were obtained after an electronic search on various scientific databases including PubMed, Scopus, and Web of Science. This comprehensive search included words such as wound healing, foreign body response, silicone implants, adsorption, inflammation, and fibrosis. The authors further screened the identified manuscripts to meet the inclusion criteria. Duplicate and non-English manuscripts were removed.

## 3 Wound healing response to tissue injury

The implantation of a silicone breast implant inevitably causes tissue injury, initiating a wound healing response that shares key features with the FBR (Gonzalez et al., 2016; George et al., 2006). Both processes involve a complex interplay of cells, cytokines, and extracellular matrix (ECM) components (Gonzalez et al., 2016; George et al., 2006). Wound healing progresses through distinct phases: hemostasis, inflammation, proliferation, and remodeling (Rodrigues et al., 2019). Initially, hemostasis stems bleeding and establishes a provisional matrix for cell migration (Furie and Furie, 2008; Skover, 1991; Schultz et al., 2011; Periayah et al., 2017; Junker et al., 2013; Sieggreen, 1987). Platelets are crucial, releasing growth factors like transforming growth factor-beta (TGF-β), epidermal growth factor (EGF), and platelet-derived growth factor (PDGF) that stimulate subsequent phases (Senzel et al., 2009; Amable et al., 2013). Inflammation then recruits immune cells to the injury site (Mittal et al., 2014; Bergamini et al., 2004; Ludes et al., 2021; Soliman and Barreda, 2022), which eliminate pathogens and orchestrate the proliferative phase (He and Marneros, 2013; Delavary et al., 2011; Shapouri‐Moghaddam et al., 2018; Krzyszczyk et al., 2018; Novak and Koh, 2013). During proliferation, fibroblasts synthesize new ECM and endothelial cells form new blood vessels (Hosgood, 2006; Strodtbeck, 2001; Johnson and Wilgus, 2014; Bainbridge, 2013; Lin et al., 2023). Finally, remodeling leads to scar formation, marked by a shift in collagen composition and wound contraction (Haukipuro et al., 1991; Darby et al., 2014; Gurtner et al., 2008; Gill et al., 2003; Telgenhoff and Shroot, 2005). Understanding wound healing provides a foundation for comprehending the FBR, as both share fundamental mechanisms and involve a complex interplay of cellular and molecular events.

### 3.1 Hemostasis

The wound healing process is initiated by hemostasis, a critical step involving the rapid cessation of bleeding from damaged blood vessels (Furie and Furie, 2008; Skover, 1991). This process is driven by vasoconstriction, narrowing the blood vessels, and platelet activation (Schultz et al., 2011). Platelets aggregate at the site of injury, forming a plug in response to exposed subendothelial collagen (Periayah et al., 2017). Activated platelets also release factors that promote the deposition of fibrinogen, which is then converted to insoluble fibrin strands, further reinforcing the platelet plug (Periayah et al., 2017). This combined structure forms a thrombus, effectively sealing the ruptured vessels and preventing further blood loss (Junker et al., 2013; Sieggreen, 1987). Importantly, the fibrin network also serves as a provisional matrix, providing a scaffold for the migration of other cells crucial for subsequent stages of wound healing (Junker et al., 2013; Sieggreen, 1987). Furthermore, platelets embedded within the thrombus release a variety of biomolecules, including TGF-β, EGF, and PDGF, which stimulate and orchestrate the subsequent phases of wound healing, including inflammation and proliferation (Senzel et al., 2009; Amable et al., 2013). This highlights the multifaceted role of platelets in hemostasis, extending beyond clot formation to actively modulate the overall wound healing response.

### 3.2 Inflammation

The inflammatory phase of wound healing is a critical stage orchestrated by a complex network of cellular and molecular signals. It is initiated by the release of various mediators from injured cells, including reactive oxygen species (ROS), damage-associated molecular patterns (DAMPs), bioactive lipids, and cytokines/chemokines (Mittal et al., 2014; Bergamini et al., 2004; Ludes et al., 2021; Soliman and Barreda, 2022). These signals act as distress beacons, alerting the immune system to tissue damage and initiating an inflammatory response.

Resident cells, including mast cells and macrophages, are quick to respond to these signals, becoming activated and releasing additional inflammatory mediators that amplify the response and recruit circulating immune cells to the injury site (He and Marneros, 2013). Neutrophils, the first line of defense, rapidly infiltrate the wound, acting as phagocytic sentinels that engulf pathogens and prevent infection (Delavary et al., 2011). Following neutrophil infiltration, monocytes infiltrate the wound and differentiate into macrophages, further bolstering the immune response (Shapouri‐Moghaddam et al., 2018). Macrophages are highly versatile and orchestrate inflammation and subsequent stages of wound healing. They not only eliminate pathogens through phagocytosis and the production of antimicrobial substances but also clear cellular debris and release signaling molecules that regulate tissue repair (Krzyszczyk et al., 2018).

Macrophages exhibit remarkable plasticity and dynamically adapt to their phenotype and function in response to environmental cues (Novak and Koh, 2013). Early in the inflammatory phase, pro-inflammatory M1 macrophages predominate, driving the immune response against pathogens (Krzyszczyk et al., 2018). As the wound healing process progresses, there is a shift towards a predominance of M2 macrophages, which promotes tissue repair and resolution of inflammation (Novak and Koh, 2013). This phenotypic switch is essential for efficient wound healing and transition to the subsequent proliferative phase.

### 3.3 Proliferation

The proliferative phase marks a turning point in wound healing, shifting the focus from defense to reconstruction (Hosgood, 2006). This phase is characterized by a surge in cellular activity, with various cell types playing crucial roles in rebuilding damaged tissues. Epithelial cells such as keratinocytes proliferate, migrate, and differentiate to restore the epidermal barrier and effectively seal the wound (Strodtbeck, 2001). In deeper tissues, such as the dermis and hypodermis, the activity of endothelial cells and fibroblasts is at the central stage (Johnson and Wilgus, 2014). Endothelial cells, the architects of blood vessels, proliferate and migrate to form new vascular networks via angiogenesis (Bainbridge, 2013). This process is crucial for supplying the regenerating tissue with oxygen and nutrients, which are necessary for repair. Simultaneously, fibroblasts within the wound, the master builders of the ECM, proliferate, and begin synthesizing new ECM components (Bainbridge, 2013). This new ECM provides structural support and a scaffold for tissue regeneration.

As the proliferative phase progresses, granulation tissue emerges, replacing the initial fibrin clot (Lin et al., 2023). This nascent tissue, rich in collagen and newly formed blood vessels, serves as a foundation for the final scar tissue matrix (Lin et al., 2023). The proliferative phase, therefore, represents a critical bridge between the initial inflammatory response and the final remodeling stage, laying the groundwork for tissue regeneration and scar formation.

### 3.4 Remodeling and scar formation

The final remodeling phase is a protracted yet essential process that transforms the initial granulation tissue into a mature, relatively avascular scar (Haukipuro et al., 1991; Darby et al., 2014). This phase is characterized by extensive ECM remodeling, orchestrated primarily by fibroblasts, the key cellular players in this stage. A hallmark of the remodeling phase is the shift in collagen composition within the scar tissue. The initial collagen III-rich ECM, characteristic of granulation tissue, is gradually replaced by a more robust and organized ECM rich in collagen I (Haukipuro et al., 1991; Darby et al., 2014). This transition is driven by the increased synthesis of collagen I and the concurrent degradation of collagen III by enzymes known as matrix metalloproteinases (MMPs) (Gurtner et al., 2008).

The delicate balance between MMPs, responsible for ECM degradation, and tissue inhibitors of metalloproteinases (TIMPs), is crucial for proper scar formation (Gill et al., 2003; Telgenhoff and Shroot, 2005). A disruption of this balance, particularly an overabundance of MMP activity, can lead to excessive ECM breakdown and impaired scar formation, while an imbalance favoring TIMPs can result in excessive scar tissue formation (Gill et al., 2003; Telgenhoff and Shroot, 2005). Myofibroblasts, specialized contractile fibroblasts, play key roles during the remodeling phase. These cells generate significant contractile forces, facilitating wound closure and contributing to the overall organization and strength of the scar tissue. However, the persistent presence or abnormal clearance of myofibroblasts can lead to excessive ECM deposition and contribute to pathological scarring (Telgenhoff and Shroot, 2005). Therefore, the tightly regulated activity of fibroblasts and myofibroblasts, coupled with the balanced interplay between MMPs and TIMPs, is crucial for achieving optimal scar formation and tissue regeneration.

## 4 Foreign body response to implants

The body’s response to a silicone breast implant mirrors many aspects of normal wound healing, yet with distinct consequences (Kyriakides and Bornstein, 2003). The surgical procedure itself causes tissue damage, eliciting a wound-healing-like response (Kyriakides and Bornstein, 2003). However, the presence of the implant as a foreign object triggers a unique cascade of events known as the FBR (Noskovicova et al., 2021a). This response, while sharing similarities with wound healing, ultimately isolate the implant from the host tissue by encapsulating it within a fibrous capsule (Noskovicova et al., 2021a). In some cases, this process can become dysregulated, leading to excessive fibrosis and complications such as capsular contracture (Noskovicova et al., 2021a).

The FBR to silicone implants typically progresses through five key phases: 1 protein adsorption and provisional matrix formation, 2 acute inflammation, 3 chronic inflammation, 4 foreign body giant cell formation, and 5 encapsulation (Table 1). This complex process involves a dynamic interplay of various cells and the extracellular matrix, ultimately shaping the long-term fate of the implant.

**TABLE 1:** The five key stages of FBR, the cell types, cellular interactions and molecular interactions involved.

Stage of FBR (time course)	Cell types involved	Cellular interactions	Molecular interactions
Protein Adsorption (seconds to minutes)	None (acellular initial phase); sets stage for leukocytes	Rapid, non-specific binding of plasma proteins to implant surface; Vroman effect dictates sequential displacement	Albumin, fibrinogen, fibronectin, vitronectin, complement (e.g., C3b), γ-globulin adsorb; integrins mediate future cell adhesion; complement activation initiates coagulation-inflammation cross-talk
Acute Inflammation (hours to days)	Neutrophils, monocytes, macrophages, mast cells, platelets	Neutrophil migration and degranulation; monocyte differentiation to macrophages; mast cell histamine release recruits phagocytes; platelet activation aids clot formation and cell recruitment	Release of ROS, proteolytic enzymes, chemokines (e.g., CXCL4, LTB4), cytokines (TNF-α, IL-1β, IL-6, IL-8); β2 integrins (αMβ2) bind fibrinogen/fibronectin; TGF-β, PDGF from platelets
Chronic Inflammation (days to weeks)	Macrophages (M1 to M2 shift), lymphocytes, fibroblasts (early)	Macrophage adhesion and activation; lymphocyte-macrophage cross-talk sustains response; frustrated phagocytosis as cells fail to degrade implant	Pro-inflammatory cytokines (TNF-α, IL-1, IL-6); chemokines (CCL2/MCP-1, CCL3/MIP-1α); transition to anti-inflammatory IL-10, TGF-β; β1/β2 integrins for adhesion; MMPs regulate ECM remodeling
FBGC Formation (weeks to months)	Macrophages, FBGCs	Macrophage fusion into multinucleated FBGCs; persistent surface adhesion; attempted engulfment/degradation	IL-4, IL-13 induce fusion; mannose receptor, DC-STAMP, CD47 upregulation; ROS, acid, enzymes released; vitronectin, osteopontin modulate process; rac1 signaling
Fibrous Encapsulation (months to years)	Fibroblasts, myofibroblasts, macrophages, endothelial cells	Fibroblast proliferation and transdifferentiation to myofibroblasts; macrophage-fibroblast signaling; angiogenesis for capsule vascularization; collagen deposition isolates implant	TGF-β, PDGF drive fibroblast activation; VEGF for angiogenesis; collagen (types I/III), fibronectin in ECM; IL-10, TGF-β promote resolution or fibrosis

### 4.1 Protein adsorption and provisional matrix formation

**FIGURE 2:**
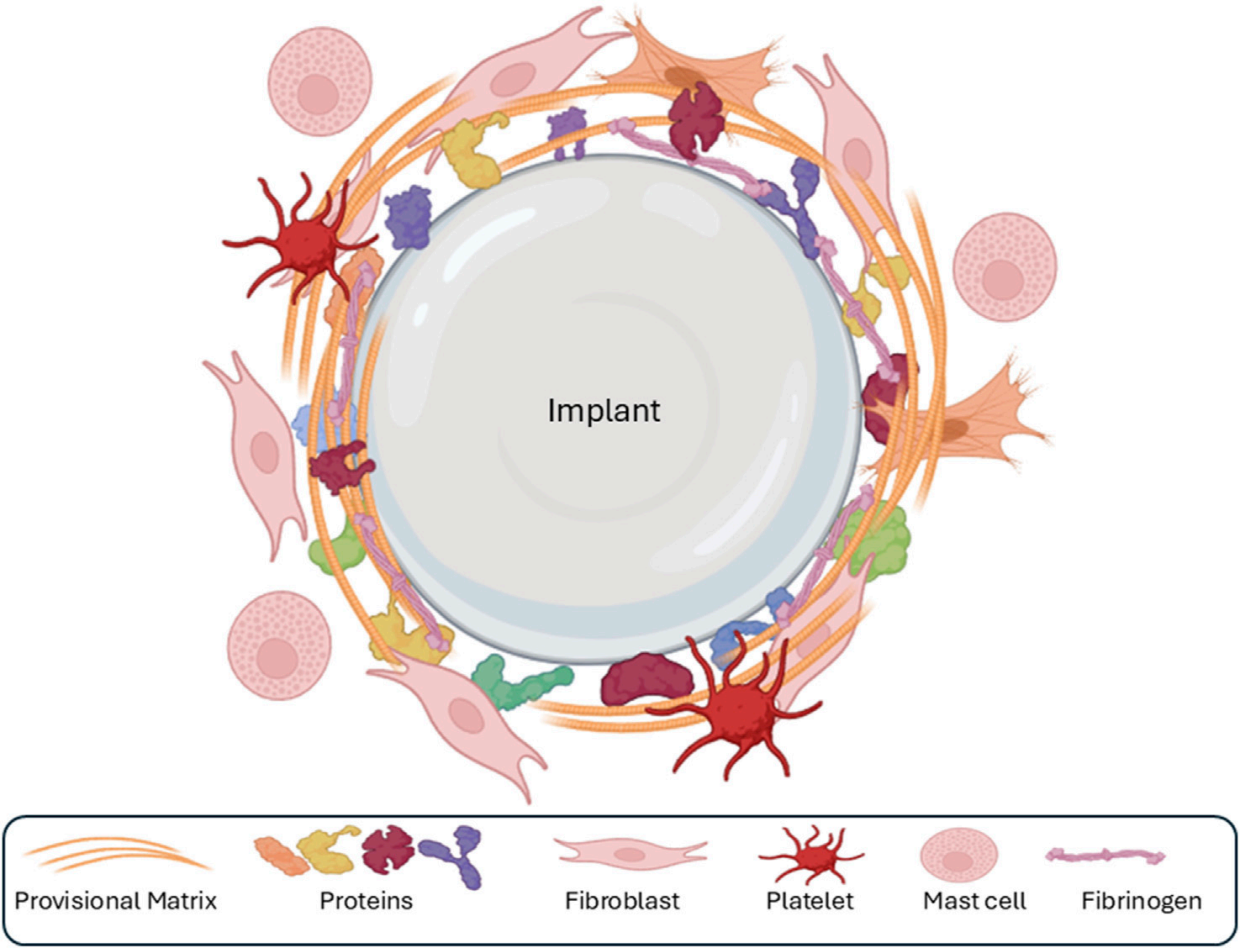
The first phase of the foreign-body response is the adsorption of various ECM components and proteins (collagens, fibronectin, fibrin, etc.) onto the surface of the implant, followed by the formation of a sparse, fibrin-rich provisional matrix around the implant.

The implantation of a silicone breast implant invariably disrupts vascularized connective tissue, initiating a wound healing response characterized by the adsorption of various blood plasma proteins onto the implant surface (Rivera-Chacon et al., 2013). These proteins, including albumin, fibrinogen, and vitronectin, exhibit high affinity for the implant material and form a fibrin-dominated provisional ECM (Rivera-Chacon et al., 2013; Figure 2). This nascent ECM, adhering to the implant surface as a 2–5 nm layer, serves as a dynamic scaffold that influences the subsequent FBR (Zhang et al., 2013). It provides structural support for infiltrating cells and acts as a reservoir for various bioactive molecules, such as mitogens, chemoattractants, cytokines, and growth factors, which are continuously released and modulate the FBR (Wells et al., 2017).

The composition and structure of this initial protein layer are critical, as they can significantly influence long-term outcomes. For instance, an excessively thick protein layer or specific protein conformations that promote cell adhesion may predispose to excessive capsule formation and fibrosis around the implant. Protein adsorption onto biomaterials is a dynamic process involving several mechanisms, including adsorption and desorption, competitive exchange, and exchange through transient complex formation (Darby et al., 2014; Gurtner et al., 2008). The complexity of this process *in vivo*, involving numerous proteins and dynamic interactions, remains an area of active investigation.

The initial phase of protein adsorption is often governed by the Vroman effect, characterized by the sequential adsorption of proteins based on their size and mobility (Noh and Vogler, 2007). Smaller, more mobile proteins, like albumin, are initially adsorbed but are subsequently replaced by larger and adhesive proteins on the implant surface (Kim and Yoon, 2002; Horbett, 2018; Voskerician et al., 2000). This dynamic exchange is particularly prominent on hydrophilic surfaces, as protein binding is less tenacious compared to hydrophobic surfaces (Noh and Vogler, 2007; Kim and Yoon, 2002). Ultimately, the final protein composition on the implant surface is determined by a complex interplay of factors, including serum protein concentrations, surface characteristics of the implant material, and the individual protein properties (Voskerician et al., 2000).

The specific proteins adsorbed onto the implant surface play distinct roles in the subsequent FBR. Vitronectin and fibronectin, for example, are critical for monocyte adhesion to the provisional matrix and the implant surface (Shen et al., 2004). Fibrinogen, in addition to its role in coagulation (Gill et al., 2003), also promotes the adhesion of platelets, neutrophils, and macrophages, thereby influencing subsequent inflammatory phases of the FBR (Darby et al., 2014; Gurtner et al., 2008). Understanding the intricacies of protein adsorption and the dynamic interplay of adsorbed proteins is crucial for comprehending the FBR and developing strategies to modulate this response for improved implant biocompatibility.

For silicone implants inserted into the breast socket, protein adsorption, governed by the Vroman Effect, occurs immediately upon contact with blood and interstitial fluids, forming a provisional matrix that dictates the subsequent cellular responses (Richter-Bisson and Hedberg, 2025). The design strategies for silicone implants should prioritize the modulation of early protein adsorption to foster biocompatibility. Inhibiting fibrinogen adsorption is paramount because its conformational changes expose epitopes that recruit macrophages and trigger proinflammatory cascades, exacerbating FBR and capsular contracture (Jung et al., 2003). Similarly, suppressing unfolded immunogenic proteins such as albumin prevents denaturation-induced inflammation and immune activation (Ballet et al., 2010). Conversely, promoting fibronectin adsorption enhances integrin-mediated cell adhesion, facilitating extracellular matrix remodeling and tissue integration, thereby mitigating fibrosis.

Surface modifications, such as zwitterionic coatings (for example, poly(2-methacryloyloxyethyl phosphorylcholine) (MPC)) or polyethylene glycol (PEG) grafting), can selectively reduce fibrinogen binding while favoring fibronectin binding, achieved via increased hydrophilicity and antifouling properties (Fischer et al., 2018). This initial adsorption event is critically important, as it orchestrates all downstream immune and fibrotic responses, including macrophage fusion, cytokine release (e.g., TGF-β), and collagen deposition, potentially leading to complications such as contracture or implant failure. Controlling early protein adsorption offers a foundational approach to enhance long-term outcomes. Several studies have demonstrated the benefits of doing so. Kang et al. (2020) demonstrated MPC-coated silicone implants reduced protein adsorption by 55%–64%, yielding thinner capsules and lower inflammation in porcine models (Kang et al., 2020). Zeplin et al. (2010) showed halofuginone coatings inhibited fibrinogen-driven fibrosis, decreasing capsule thickness and TGF-β levels in rats (Zeplin et al., 2010). Kim et al. (2020) used PEG-linked liposomes containing phosphatidylserine to minimize fibrinogen and enhance fibronectin-like integration, reducing fibrous encapsulation (Kim et al., 2020). These interventions underscore the potential of adsorption control in improving implant durability and patient safety.

#### 4.1.1 Implant surface properties modulation of protein adsorption and provisional matrix formation

The physicochemical properties of an implant surface significantly influence the initial protein adsorption process, ultimately shaping the composition and structure of the provisional matrix and the subsequent FBR (Foroushani et al., 2022; Lam et al., 2021). Surface topography, wettability, and even tensile strength play crucial roles in determining the type and amount of proteins adsorbed (Foroushani et al., 2022; Lam et al., 2021). Furthermore, the plasma concentration of individual proteins and their inherent structural characteristics also contribute to their adsorption profiles (Wilson et al., 2005).

Interestingly, while the initial protein adsorption patterns are critical, they do not fully predict the final composition of the provisional matrix (Jenney and Anderson, 2000; Horbett, 1993; Love and Jones, 2013). This highlights the dynamic and complex nature of protein interactions at the biomaterial interface. For instance, while increased surface roughness and hydrophilicity generally enhance protein adsorption (Horbett, 2018; Lee and Ruckenstein, 1988; Guha and Subramanian, 2011), the relationship between hydrophobicity and protein adsorption is not always straightforward. Although hydrophilic surfaces might initially repel proteins due to the formation of a water barrier (Wahlgren and Arnebrant, 1991; Raffaini and Ganazzoli, 2010), some studies suggest that both hydrophilic and hydrophobic surfaces can exhibit similar protein adsorption capacities (Fabre et al., 2018; Jeyachandran et al., 2009).

This complexity is further underscored by the influence of specific polymer coatings on protein adsorption. PEG, for example, is known to reduce protein adsorption, with its effectiveness correlating with chain density (Malmsten et al., 1998; Sun et al., 2014). Other polymers employed for their protein-repelling properties include oligoethylene glycol (Li et al., 2007), polyacrylamide (Xue et al., 2012; Liu et al., 2012), polycarboxybetaine methacrylate (Zhang et al., 2008), and peptoids (Mahmoudi et al., 2017).

Beyond surface properties, protein characteristics, such as conformation and charge, also play crucial roles in adsorption (Mitra, 2020; Hasan et al., 2018). Proteins like vitronectin exhibit preferential binding to charged surfaces (Li et al., 2020; Banovac et al., 1994). Moreover, surface roughness can influence protein conformation upon adsorption, potentially altering their biological activity and interactions with cells (Prasad et al., 2010; Lord et al., 2010; Le et al., 2013). This effect may be attributed to the impact of roughness on surface wettability, which in turn influences protein interactions (Vogler, 1998; MacDonald et al., 1998).

The interplay between implant surface properties and protein characteristics orchestrates a complex adsorption process that ultimately shapes the provisional matrix and influences subsequent stages of the FBR. Understanding these intricate relationships is crucial for designing biocompatible implant materials that can modulate the FBR and promote successful tissue integration.

### 4.2 Acute inflammation

**FIGURE 3:**
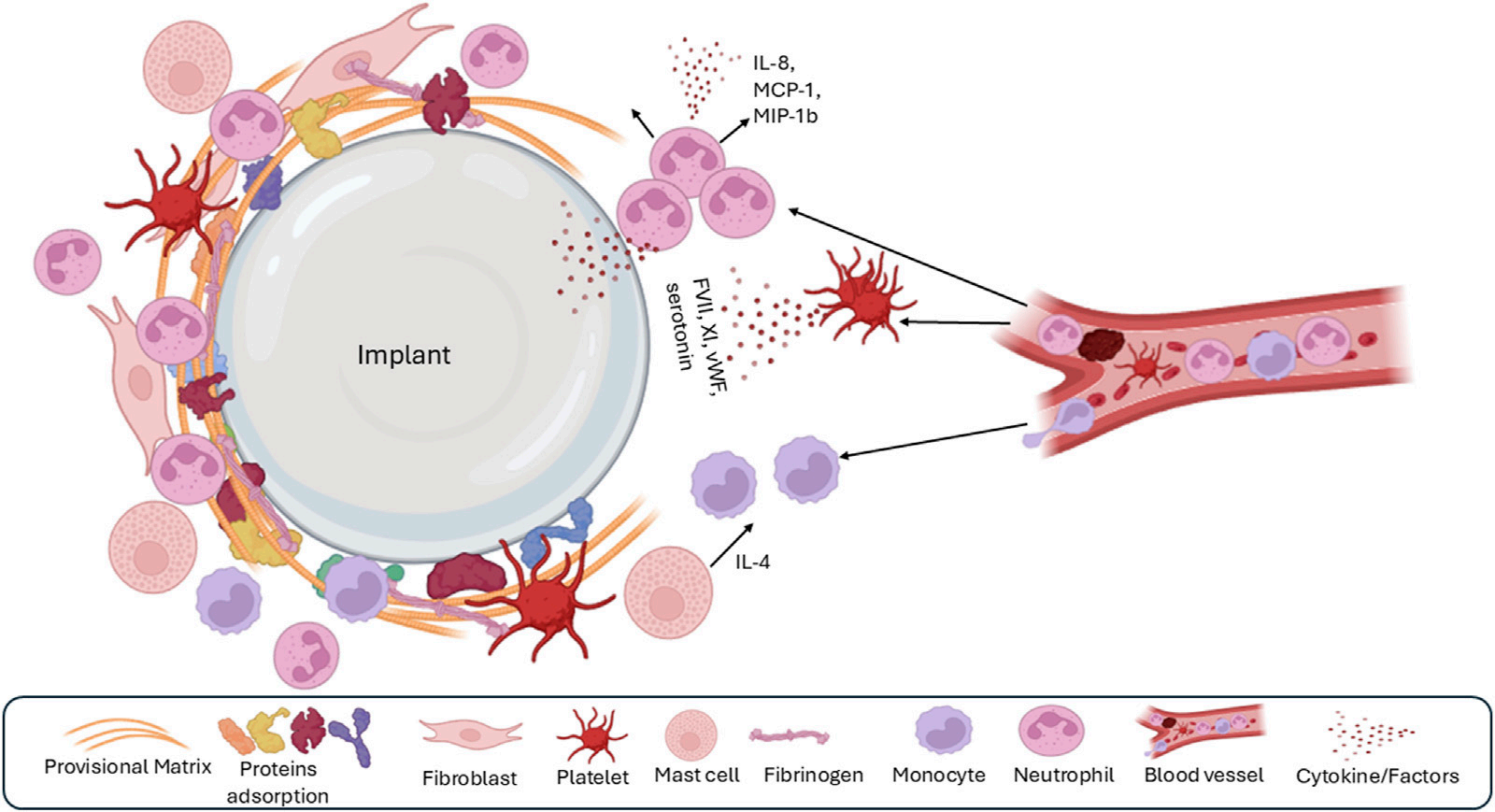
The second phase of the foreign body response is acute inflammation, which is characterized by the dominant presence of polymorphonuclear leukocytes, recruitment of monocytes and macrophages, and mast cell degranulation.

Acute inflammation is a rapid and transient response to tissue injury and the presence of a foreign body, such as a silicone implant. This phase, occurring within minutes to hours of implantation, is characterized by the orchestrated recruitment of inflammatory cells to the tissue-implant interface (Zdziennicka et al., 2021; Kizhakkedathu and Conway, 2022; Figure 3). The initial trigger for this acute inflammatory response is the tissue damage incurred during the surgical implantation procedure itself (Javdani et al., 2022; Zhou and Groth, 2018). This damage leads to the release of various biomolecules, including lipids, ATP, and heat shock proteins, which act as “danger signals” alerting the immune system to the injury (Kono and Rock, 2008). These danger signals, along with factors released from activated platelets such as platelet factor IV, initiate the recruitment of leukocytes, primarily neutrophils, to the implant site (Boni et al., 2019; Jhunjhunwala, 2017). Neutrophils are the first responders, rapidly migrating to the site of injury and playing a critical role in eliminating potential pathogens and preventing infection (Boni et al., 2019; Ellis et al., 2018). The provisional matrix also contributes to leukocyte activation and recruitment (Barker and Engler, 2017; Modulevsky et al., 2016; Klopfleisch and Jung, 2017). Mast cells in the surrounding tissue further amplify the inflammatory response by releasing histamine and serotonin, which induce vasodilation and increase vascular permeability, facilitating the influx of inflammatory cells to the implant site (Zdolsek et al., 2007). Mast cells also release cytokines like IL-4 and IL-13, which play a crucial role in recruiting monocytes and promoting their differentiation into macrophages (Janeway and Medzhitov, 2002).

Macrophages, the central orchestrators of the FBR, become a prominent cell population at the implant site, phagocytosing cellular debris, damaged tissue, and potential implant degradation products (Kenneth Ward, 2008). The activation of complement products and the potential presence of bacteria further contribute to the inflammatory milieu by generating chemoattractants that attract additional leukocytes (Kyriakides and Bornstein, 2003; Noskovicova et al., 2021a; Rivera-Chacon et al., 2013; Zhang et al., 2013; Labow et al., 2001; De Filippo et al., 2013).

This complex interplay of cellular and molecular events creates a highly pro-inflammatory microenvironment at the tissue-implant interface, driving further leukocyte recruitment and setting the stage for the subsequent chronic inflammatory phase (Kanterman et al., 2012; Wang et al., 2022). The acute inflammatory phase, while typically transient, is crucial in shaping the overall FBR and can significantly influence the long-term outcome of implant integration.

#### 4.2.1 Implant surface properties modulation of acute inflammation

While the acute inflammatory phase is transient, the impact of implant surface properties on this stage can have profound implications for the subsequent progression of the FBR. Although research on this specific phase is limited due to its short duration, several key factors have been identified.

The extent of tissue damage during implant insertion is a major determinant of the severity of acute inflammation (Chang and Merritt, 1994). Greater surgical trauma leads to increased release of danger signals and a more robust inflammatory response. Similarly, the amount and composition of the initial protein adsorption layer influence the activation and recruitment of immune cells (Chang and Merritt, 1994).

Surface properties play a role in modulating the acute inflammatory response. Increased surface roughness, while potentially promoting protein adsorption, can also increase the risk of bacterial infection, further exacerbating inflammation (Chang and Merritt, 1994). The provisional matrix itself can modulate the inflammatory response by acting as a physical barrier, influencing the interactions between infiltrating immune cells (Lewis et al., 2014; Carnicer-Lombarte et al., 2021). Furthermore, the implant surface can directly interact with immune cells via pattern recognition receptors, such as Toll-like receptors (TLRs). For example, TLR2 and TLR4 on leukocytes can recognize the hydrophobic regions of implant surfaces, triggering immune cell activation and contributing to the inflammatory response (Carnicer-Lombarte et al., 2021).

The interaction between the provisional matrix and immune cells is also crucial for determining the transition from acute to chronic inflammation (Martin and Garcia, 2021; Babensee, 2020; Lickorish et al., 2004). Macrophages and polymorphonuclear cells interact with the provisional matrix, and their activation state can influence the duration and intensity of the inflammatory response. The release of pro-inflammatory mediators such as IL-4 and IL-13 during this phase further amplifies the immune response and contributes to the recruitment and activation of macrophages, which are key players in chronic inflammation (Demir, 2020).

While acute inflammation is a fleeting phase, the interplay between implant surface properties, the provisional matrix, and immune cell activation during this stage sets the stage for the subsequent phases of the FBR and ultimately influences the long-term outcome of implant integration.

### 4.3 Chronic inflammation

**FIGURE 4:**
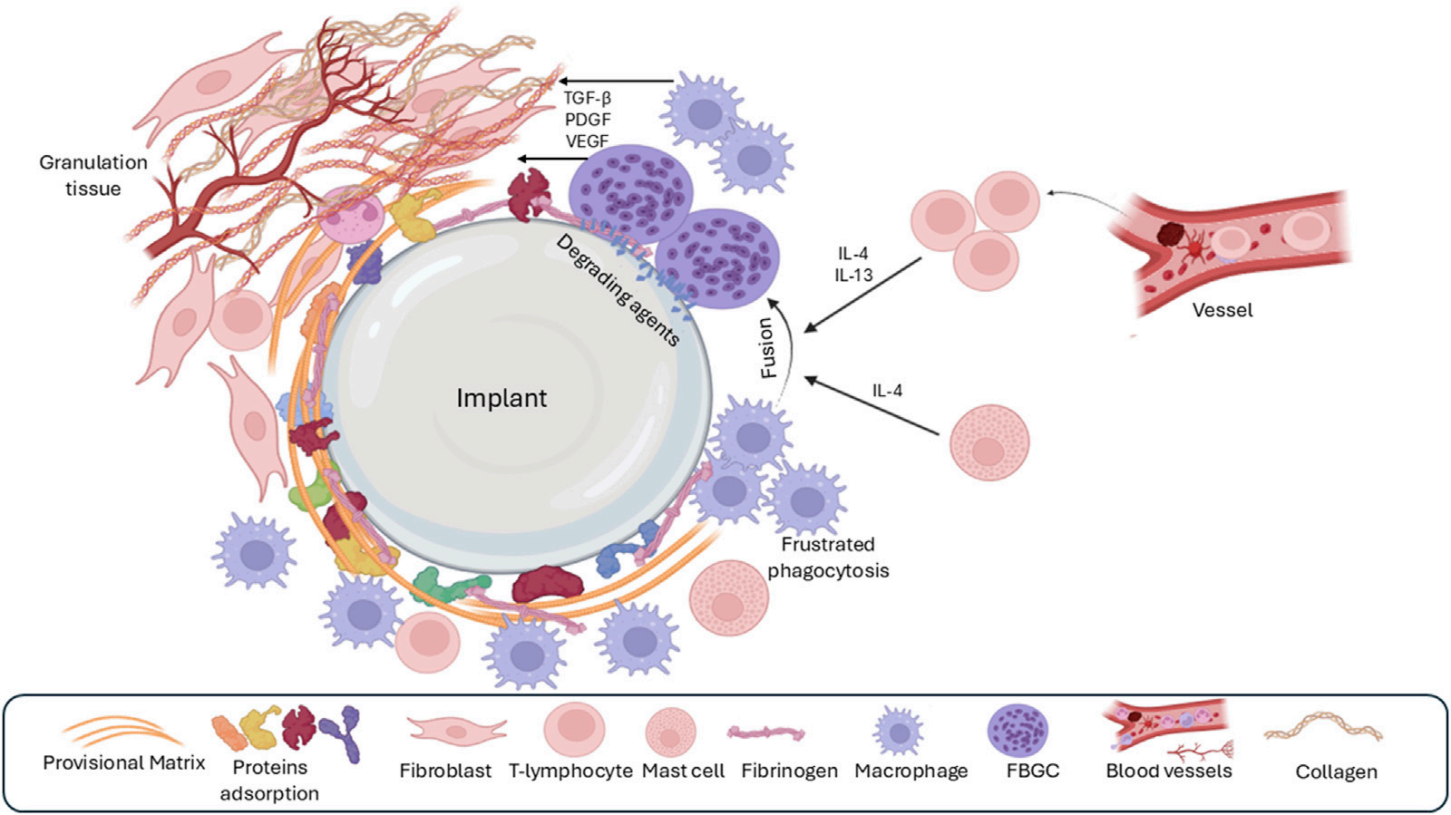
The third phase of the foreign-body response is chronic inflammation, which is characterized by the presence of many macrophages and lymphocytes around the implant. Over time, macrophages fuse to form foreign body giant cells (FBGC) because of unsuccessful phagocytosis (frustrated phagocytosis) as well as the effect of IL4 and IL13 derived from mast cells. Granulation tissue formation is a result of the release of various growth factors by macrophages and FBGC.

Chronic inflammation represents a persistent immune response that can significantly impact the long-term success of implant integration. Macrophages are the central players in this phase, orchestrating a complex network of cellular and molecular interactions (Martin and Garcia, 2021; Sheikh et al., 2015). Monocytes, recruited from the bone marrow and spleen, migrate to the implant site and differentiate into macrophages (Gerhardt and Ley, 2015; Kzhyshkowska et al., 2015; Figure 4). This recruitment is driven by various growth factors and cytokines, including macrophage inflammatory protein 1α, TGF-β, and platelet-derived growth factor (Zhao et al., 1992; McNally et al., 1996).

Upon arrival, macrophages interact with the provisional matrix, adhering to proteins like fibronectin and fibrinogen via integrin receptors (Sheikh et al., 2015; Rowley et al., 2019; Hsieh et al., 2017). This interaction is crucial for macrophage activation and polarization into distinct phenotypes with specialized functions (Sheikh et al., 2015; Rowley et al., 2019; Hsieh et al., 2017). Macrophages contribute to the vascularization of the surrounding tissue by secreting pro-angiogenic factors such as TGF-β, PDGF, and vascular endothelial growth factor (VEGF) (Xu et al., 2013). They also release a diverse array of chemokines, cytokines, and other signaling molecules that modulate the inflammatory microenvironment and influence the progression of the FBR (Xu et al., 2013).

A key factor contributing to the transition from acute to chronic inflammation is the “frustrated phagocytosis” phenomenon (Luttikhuizen et al., 2006). Macrophages attempt to engulf the implant but are unable to do so due to its size or material properties, leading to their persistent activation and the perpetuation of the inflammatory response (Luttikhuizen et al., 2006).

Chronic inflammation also involves the infiltration of lymphocytes, which further modulates the immune response. T lymphocytes release cytokines like IL-4 and IL-13, which promote the polarization of macrophages from the pro-inflammatory M1 phenotype to the pro-healing and pro-fibrotic M2 phenotype (Major et al., 2015; Kzhyshkowska et al., 2015; Mariani et al., 2019; Pinhal Enfield and Leibovich, 2011). M2 macrophages are key to tissue remodeling and contribute to the formation of foreign body giant cells (FBGCs) (Major et al., 2015; Kzhyshkowska et al., 2015; Mariani et al., 2019; Pinhal Enfield and Leibovich, 2011).

While the M1/M2 paradigm provides a useful framework for understanding macrophage function, it is important to recognize that macrophages exist along a spectrum of activation states, with various intermediate phenotypes exhibiting diverse functions (Major et al., 2015; Szott and Horbett, 2011; Yu et al., 2015; Mooney et al., 2014). These different macrophage phenotypes play distinct roles in chronic inflammation and tissue regeneration. For example, M2 macrophages are associated with reduced implant biointegration and increased angiogenesis (Labow et al., 2001; Rayahin and Gemeinhart, 2017; Jackson et al., 2023), while M1 macrophages are crucial for eliminating pathogens and promoting cell recruitment (Garg et al., 2013; Sridharan et al., 2015).

Further research is ongoing to fully elucidate the complex interplay of macrophage phenotypes and their contributions to the FBR. This knowledge is crucial for developing strategies to modulate macrophage polarization and promote successful implant integration.

#### 4.3.1 Implant surface properties modulation of chronic inflammation

The chronic inflammatory phase of the FBR is significantly influenced by the physicochemical properties of the implant surface. Surface roughness, as well as the type and amount of adsorbed proteins, can modulate macrophage activation and polarization, ultimately affecting the progression of chronic inflammation (Anderson, 2015; Lv et al., 2018; Hamlet et al., 2012). Additionally, factors such as bacterial infection and implant movement within the breast pocket can exacerbate and prolong the inflammatory response (Kyriakides and Bornstein, 2003; Noskovicova et al., 2021a).

Macrophages, equipped with pattern recognition receptors like TLRs, can directly sense and respond to the implant surface (Love and Jones, 2013). This recognition triggers signaling cascades that influence macrophage activation and cytokine production. Furthermore, the surface properties of the implant can directly affect macrophage adhesion and behavior. Hydrophobic surfaces, for example, tend to enhance macrophage attachment compared to hydrophilic surfaces (Anderson, 2015; Lv et al., 2018; Hamlet et al., 2012). The presence of specific chemical groups on the implant surface, such as amino and hydroxyl groups, can also promote macrophage and lymphocyte infiltration, leading to a more pronounced chronic inflammatory response (Ion et al., 2015; Zhou et al., 2017; Jenney and Anderson, 1999).

Interestingly, the topography of the implant surface, particularly the presence of pores, can influence macrophage recruitment and polarization. Implants with pore sizes between 30 and 40 µm have been shown to promote macrophage recruitment and their activation towards the M2 phenotype (Sussman et al., 2014; Li et al., 2022; Ma et al., 2014; Zhu et al., 2021). These M2 macrophages secrete anti-inflammatory cytokines and growth factors, potentially contributing to tissue repair and resolution of inflammation.

When macrophages encounter an implant that is too large or resistant to phagocytosis, they engage in “frustrated phagocytosis,” adhering to the implant surface via podosomes rather than focal contacts (Zhang et al., 2013). This persistent interaction, coupled with the ongoing production of inflammatory cytokines like IL-4 and IL-13 by immune cells, can lead to macrophage fusion and the formation of FBGCs (Wells et al., 2017; Noh and Vogler, 2007; Kim and Yoon, 2002). Chemoattractants, such as CCL2, further contribute to this process by directing macrophages towards each other, facilitating their fusion (Horbett, 2018).

The chronic inflammatory phase of the FBR is a dynamic process influenced by a complex interplay of implant surface properties, protein adsorption, and immune cell interactions. Understanding these factors is critical for developing strategies to modulate the inflammatory response and promote successful implant integration.

### 4.4 Foreign body giant cell formation

**FIGURE 5:**
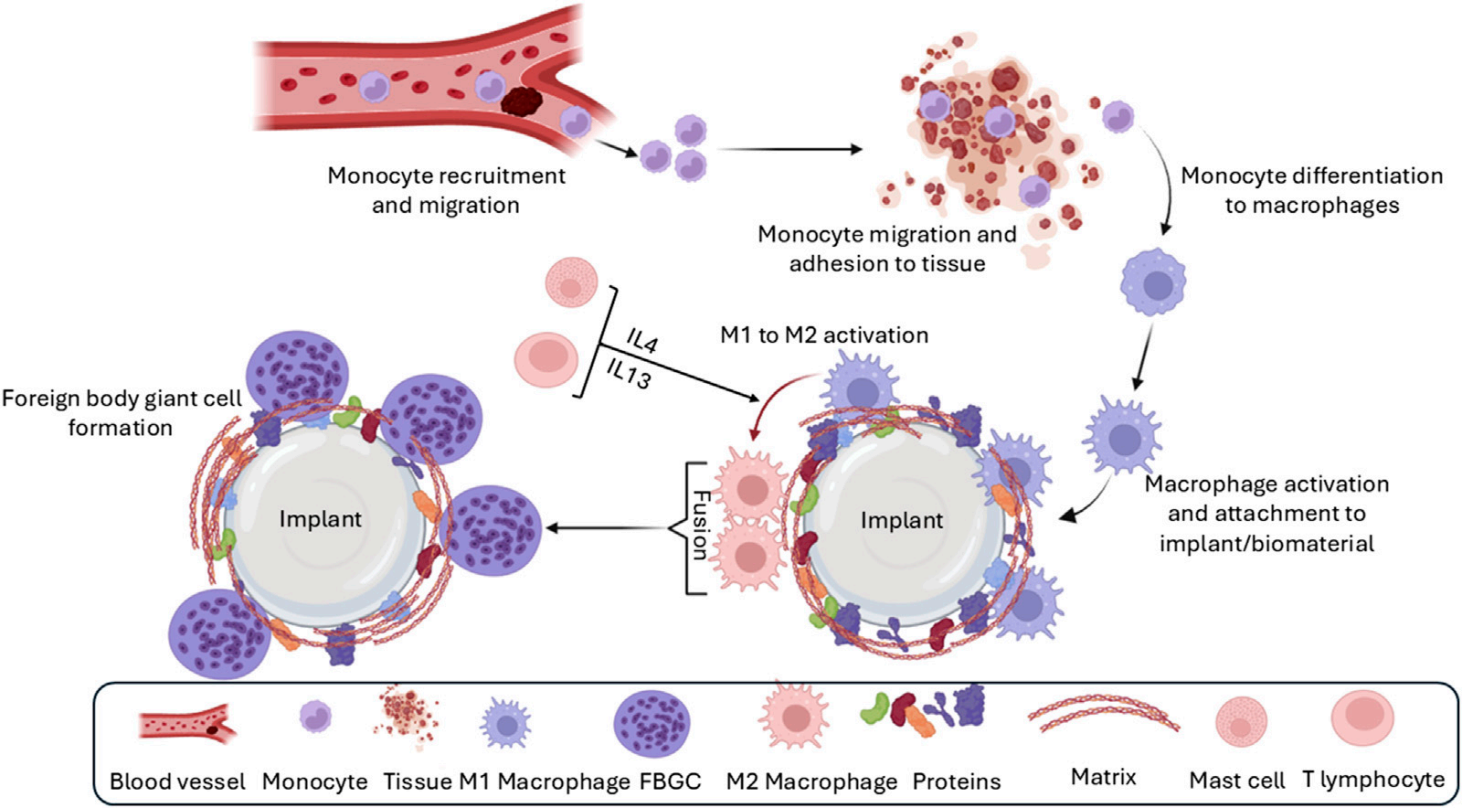
Formation of foreign body giant cells. Recruited and/or local-resident monocytes are recruited to the wound or implant site. Monocytes differentiate into M1 macrophages, polarize into M2 macrophages, and eventually fuse into foreign body giant cells (FBGCs).

The formation of FBGCs is a hallmark of the FBR, making it distinct from chronic inflammatory response (Bryers et al., 2012). These multinucleated giant cells arise from the fusion of macrophages in an attempt to engulf the implant, a process often triggered by “frustrated phagocytosis” when macrophages encounter an implant too large to internalize (Zhang et al., 2013; Smetana, 1987; Ahmadzadeh et al., 2022; McNally and Anderson, 2011). FBGCs are imposing structures, measuring several hundred micrometers in size and containing numerous nuclei (Figure 5; Zhang et al., 2013; Smetana, 1987). Once formed, they persist at the implant site as long as the implant is present (Brodbeck and Anderson, 2009). While the precise role of FBGCs in the FBR remains an area of ongoing research, their formation is thought to represent a mechanism to enhance phagocytic efficiency or potentially evade apoptosis (Ahmadzadeh et al., 2022; McNally and Anderson, 2011).

The interaction between macrophages and implants, and thus the likelihood of FBGC formation (Figure 5), is influenced by various factors, including the size of the implant or its fragments. Plasma proteins adsorb onto biomaterials, forming biomaterial-associated molecular patterns (BAMPs) that facilitate macrophage adhesion via integrins, notably β1 and β2 subunits, which link to the actin cytoskeleton via talin, vinculin, paxillin, and focal adhesion kinase (FAK) (Zaveri et al., 2014). This adhesion triggers mechanosensing and mechanotransduction, generating traction forces via actin polymerization and myosin II, thereby promoting haptotaxis and cell migration (Eslami-Kaliji et al., 2023). Rho-family GTPases such as Rac1 and Cdc42 regulate lamellipodia and filopodia formation, which are essential for cell protrusion and contact (Hoon et al., 2016). Macrophages readily engulf and degrade smaller particles through phagocytosis and intracellular lysosomal degradation (Table 2, second column) (Bryers et al., 2012). For larger particles (10–100 µm), macrophages fuse to form giant cells that collectively engulf and digest the material (Table 2, third column) (Bryers et al., 2012). However, when confronted with even larger implants, macrophages and FBGCs resort to extracellular digestion by releasing enzymes and lowering the pH (Table 2, fourth column) (Bryers et al., 2012). The fusion of macrophages into FBGCs is driven by a complex interplay of signals, including cytokines released by T lymphocytes and mast cells, particularly IL-4 and IL-13 (Van Dyken and Locksley, 2013; McNally and Anderson, 2002).

**TABLE 2:** Macrophage response to implants of different sizes.

Macrophage response	Macrophage-mediated phagocytosis	Giant cell-mediated engulfment	Extracellular degradation
Fragment size	<10 um	10–100 um	>100 um
Recognition	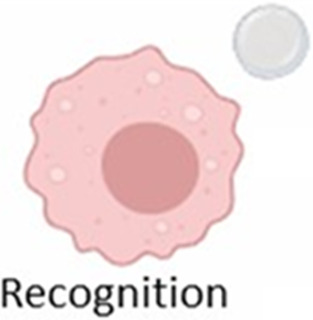	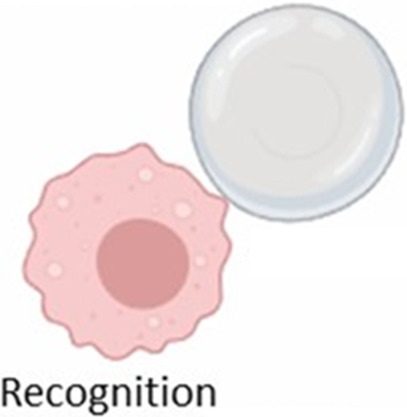	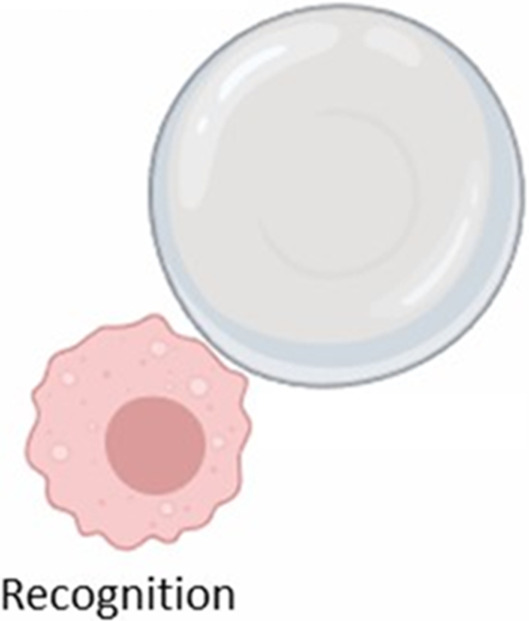
Adhesion	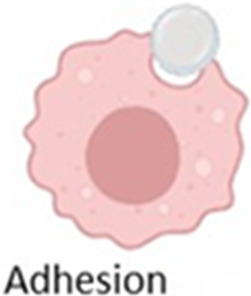	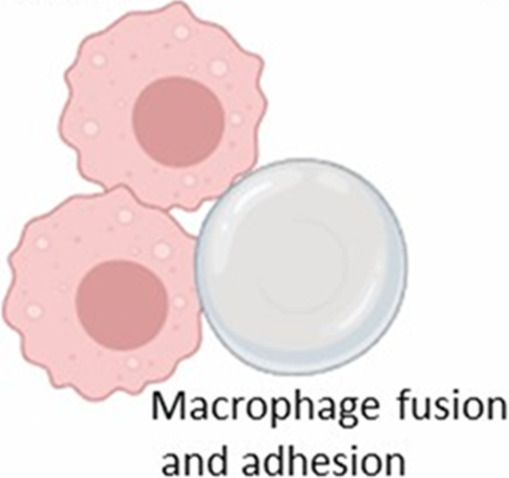	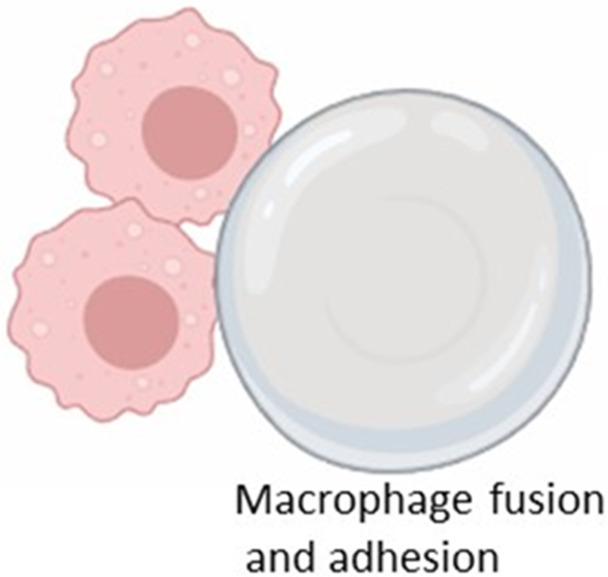
Phagocytosis	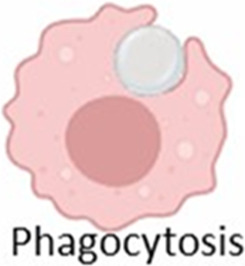	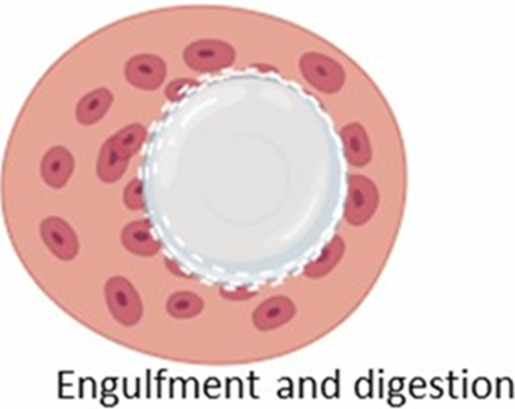	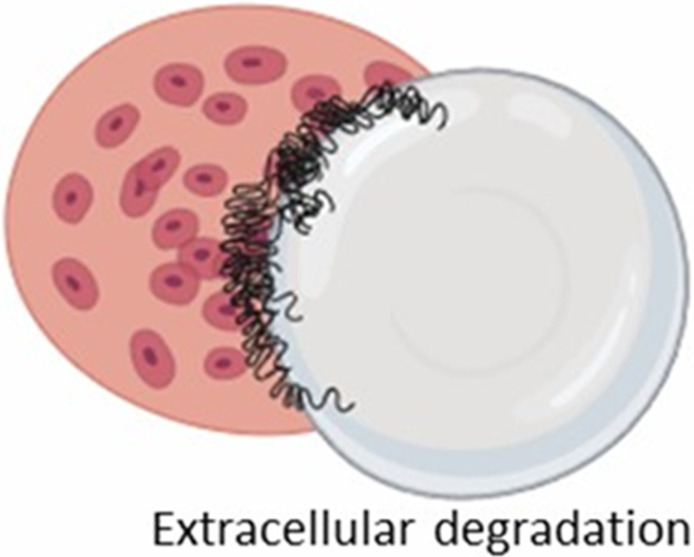
Digestion	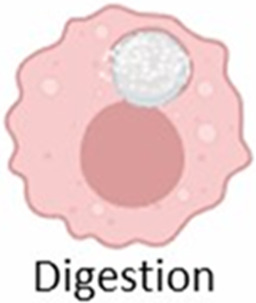		

IL-4 and IL-13 released from mast cells and T lymphocytes also activate the JAK/STAT6 pathway to upregulate fusogens such as dendritic cell-specific transmembrane protein (DC-STAMP), E-cadherin, and MMP9 (Van den Bossche et al., 2012). DC-STAMP, a seven-transmembrane protein, is indispensable for cell-cell fusion and potentially acts in a receptor-ligand manner, whereas E-cadherin enables homotypic adhesion. MMP9 is essential for extracellular matrix remodeling during fusion. Additional fusogens include the macrophage fusion receptor (SIRPα) and its ligand CD47, which inhibit phagocytosis during fusion, and CD36, which recognizes externalized phosphatidylserine (PS) on fusing membranes (Matozaki et al., 2009). Cytokines are also involved in the activation of macrophages from the pro-inflammatory M1 phenotype to the anti-inflammatory M2 phenotype, which is more prone to fusion (Shapouri‐Moghaddam et al., 2018; Braga et al., 2015; Klopfleisch, 2016; Palmer et al., 2014). The binding of lymphocytes to the implant surface via β-integrin receptors further enhances this process by upregulating the expression of cell adhesion molecules like E-cadherin, mannose receptors, and CD44 at macrophage fusion sites (McNally and Anderson, 1995; Cui et al., 2006; Han et al., 2000). FBGCs, once formed, express a variety of membrane proteins, including CD45 and CD31, and receptors for various interleukins, indicating their active participation in the immune response (Bryers et al., 2012; Anderson, 2009). They secrete a range of cytokines, including both pro-inflammatory mediators like IL-6, IL-8, and TNF-α, and anti-inflammatory cytokines like IL-10, TGF-β, and MCP-1 (Shin et al., 2018; Rashad et al., 2019).

Key signaling pathways involved in FBGC formation include DAP12/Syk, activated by M-CSF, coupled with PI3K/Akt and NF-κB to promote M2 polarization and cytoskeletal reorganization via FAK (Eslami-Kaliji et al., 2023). Purinergic signaling through P2X7 receptors detects ATP, facilitating fusion pore formation, whereas protein kinase C (PKC) isoforms β, δ, and ζ operate in diacylglycerol-dependent and -independent pathways to support cytoplasmic spreading and fusion (Lemaire et al., 2012). Podosomes and tunneling nanotubes (TNTs), which involve M-Sec and Myosin X, enable cell-cell communication and protein transfer prior to fusion (Dagar et al., 2021).

While FBGCs contribute to the isolation of the foreign material, they can also have detrimental effects. They release ROS and other bioreactive agents that can damage the implant and contribute to its degradation, potentially leading to device failure (Ahmed et al., 2016; Khan et al., 2016; Smetana et al., 2000). This degradative activity, while beneficial for resorbable materials like sutures and hydrogels (Rodriguez et al., 2009; Rizik et al., 2015; Amecke et al., 1992), is undesirable for long-term implants.

FBGC formation represents a complex and dynamic aspect of the FBR, influenced by implant characteristics, macrophage behavior, and cytokine signaling. While their role in isolating the foreign body is essential, their potential to contribute to implant degradation and chronic inflammation highlights the need for further research to fully understand their function and develop strategies to modulate their activity.

#### 4.4.1 Implant surface properties modulation of FBGC formation

The formation of FBGCs is intricately linked to the physicochemical properties of the implant surface. Macrophage fusion, the process underlying FBGC formation, is influenced by a complex interplay of factors, including cytokine signaling and the presence of membrane fusion promoters (McNally and Anderson, 2011; Kloc et al., 2022). The amount and type of proteins adsorbed onto the implant surface as well as the topographical features of the implant play crucial roles in modulating FBGC formation (Miron and Bosshardt, 2018; Neale and Athanasou, 1999).

Specific proteins within the provisional matrix, such as vitronectin and fibronectin, have been shown to directly influence FBGC formation (Collier and Anderson, 2002; Anderson et al., 1999). These proteins can modulate macrophage adhesion, activation, and subsequent fusion. Furthermore, the chemical composition of the implant surface can significantly impact FBGC formation. For instance, hydrophilic and non-ionic polyacrylic surfaces tend to reduce monocyte adhesion and differentiation into macrophages, ultimately leading to decreased FBGC formation compared to hydrophilic and cationic surfaces (Anderson, 2015).

Surface topography also exerts a significant influence on macrophage fusion and FBGC formation (Khandwekar and Rho, 2012). Studies have revealed that smooth and flat surfaces tend to promote FBGC formation compared with rough surfaces (Khandwekar and Rho, 2012). This may be attributed to the increased surface area available for macrophage adhesion and interaction on smooth surfaces, which facilitates their fusion.

The formation of FBGCs is a complex process modulated by a multitude of factors, including the presence of specific cytokines, membrane fusion promoters, adsorbed proteins, and the topographical and chemical characteristics of the implant surface. Understanding these intricate relationships is crucial for designing implant materials that can effectively modulate FBR and promote successful tissue integration.

### 4.5 Capsule formation and fibrosis

The ultimate goal of implantation is to achieve seamless biointegration of the device within the host tissue, thereby facilitating both functional restoration and tissue regeneration (Hernandez et al., 2021). However, the chronic inflammatory response elicited by the implant can result in the formation of a dense, fibrous capsule, which is a hallmark of FBR (Hernandez et al., 2021). This encapsulation process, while aimed at isolating the foreign material, can become dysregulated, resulting in excessive fibrosis and complications, such as capsular contracture.

M2 macrophages are key orchestrators of capsule formation and fibrosis (Braga et al., 2015; Zhang et al., 2021). They recruit and activate fibroblasts, promoting their differentiation into myofibroblasts, the primary producers of ECM components (Klopfleisch and Jung, 2017; Le et al., 2010). The extent of fibrosis is determined by the number of myofibroblasts and duration of their activation. In normal wound healing, the resolution of inflammation triggers myofibroblast apoptosis and a decline in collagen production, leading to scar maturation (Jun and Lau, 2010; Ramachandran et al., 2012; Bartsch et al., 2012; Kook et al., 2023). However, in the context of FBR, the persistent presence of the implant sustains a pro-inflammatory and pro-fibrotic microenvironment, preventing the resolution of fibrosis and promoting the continuous deposition of ECM (Jun and Lau, 2010; Ramachandran et al., 2012; Bartsch et al., 2012; Kook et al., 2023).

While the M1/M2 paradigm provides a simplified view of macrophage function, it is crucial to recognize the spectrum of macrophage phenotypes that exist *in vivo* (Spiller et al., 2015; Arnold et al., 2007; Mirza et al., 2013). These diverse macrophage populations contribute to fibrosis by releasing a variety of growth factors and cytokines including VEGF, TNF-α, and IL-1β (Miyagi et al., 2018; Snyder et al., 2016; Hamilton et al., 2010). Classic M2 macrophages, in particular, secrete profibrotic factors like PDGF-BB, CCL17, and CCL18, driving fibroblast activation and ECM deposition (Tarique et al., 2015; Lewis et al., 2017; Belperio et al., 2004). Recent research has shown that macrophage-myofibroblast transformation (MMT) represents a pivotal cellular plasticity event in fibrotic diseases, wherein macrophages transdifferentiate into collagen-producing myofibroblasts, exacerbating ECM deposition and tissue scarring (Ban et al., 2024). This process is tightly regulated by multiple signaling pathways and soluble factors. Central to MMT is the TGF-β/Smad pathway, where TGF-β1 binds to its receptors, activating Smad3 phosphorylation and nuclear translocation to drive expression of myofibroblast markers like α-smooth muscle actin (α-SMA) and collagen I (Zhong, 2024). Genetic ablation of Smad3 in macrophages inhibits MMT and attenuates fibrosis in models of renal and pulmonary injury (Jia et al., 2025). Complementary pathways include Wnt/β-catenin signaling, which synergizes with TGF-β to promote anti-apoptotic and pro-fibrotic phenotypes in macrophages, thereby enhancing ECM synthesis (Abaricia et al., 2020). Notch signaling, via ligands such as JAG1, also modulates MMT by stimulating α-SMA expression, while its inhibition mitigates fibrosis (Hong et al., 2019). Non-canonical regulators, such as Src tyrosine kinase activated downstream of TGF-β, are essential for MMT progression; pharmacological Src inhibition blocks this transition *in vitro* and reduces lung fibrosis *in vivo*. Cytokines such as IL-4 and IL-13 polarize macrophages toward an M2 phenotype, priming them for MMT, whereas growth factors, including PDGF and VEGF, amplify fibroblast-like functions (Noskovicova et al., 2021a). Epigenetic modifiers such as EZH2 further promote MMT via pathways such as DUSP23/Smad3 in renal models.

Emerging evidence link MMT to the FBR, which often culminates in fibrotic encapsulation and device failure (Noskovicova et al., 2021a). Direct evidence of MMT in breast implant capsules is limited and is analogous to other fibrotic contexts such as renal fibrosis, suggesting that M2-polarized macrophages transdifferentiate into myofibroblasts, contributing to dense collagen capsules. Mechanical cues from stiff implant surfaces exacerbate this, activating YAP/TAZ mechanotransduction in macrophages and fibroblasts and potentially facilitating MMT-like transitions (Tan et al., 2025).

**FIGURE 6:**
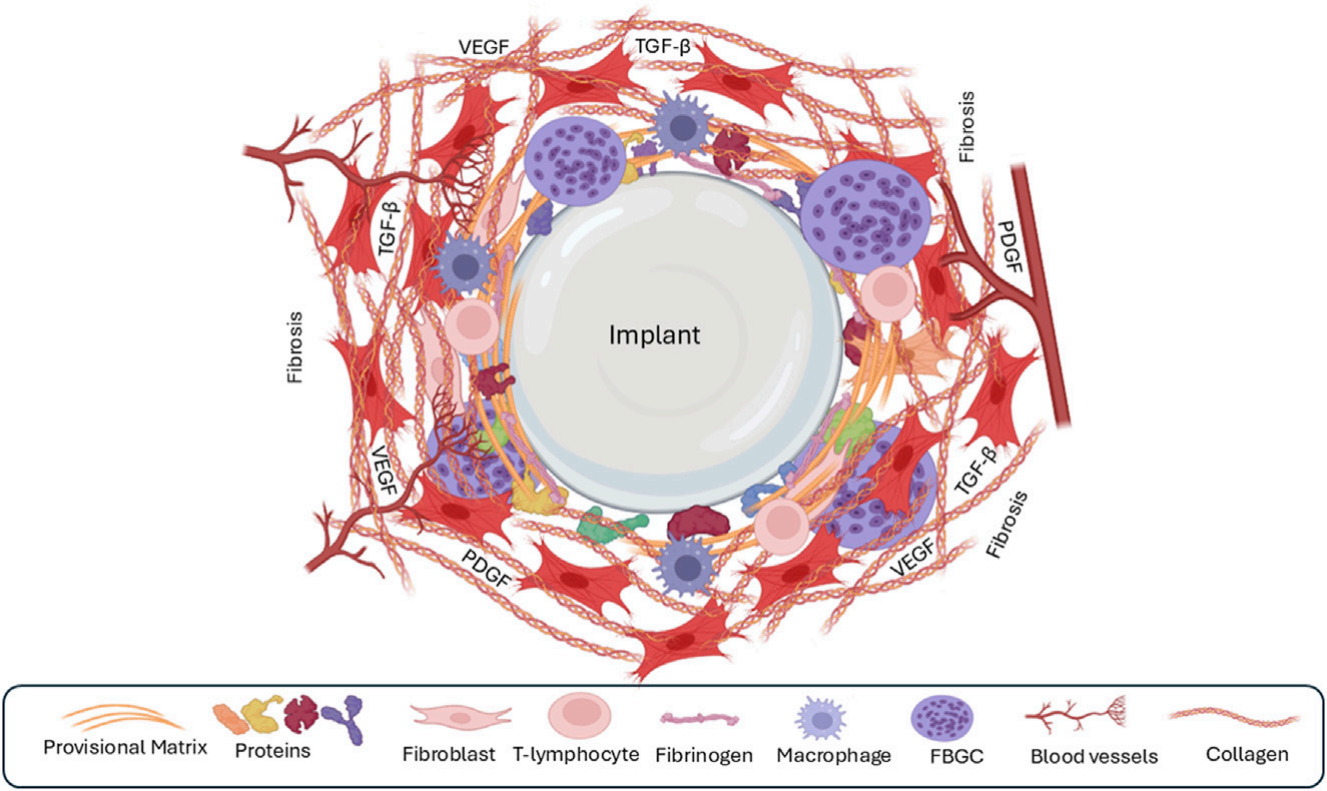
The fourth phase of the foreign-body response is the formation of a fibrous capsule around the implant. The recruitment of fibroblasts leads to the synthesis of large quantities of extracellular matrix/fibrosis, leading to the formation of a capsule around the implant. Few macrophages or other immune cells are present during this phase.

The balance between the M1 and M2 macrophages influences the extent of fibrosis. Elevated M1 activity is associated with reduced ECM deposition and heightened inflammation (Ploeger et al., 2013), while M2 macrophages promote fibrosis by inducing fibroblasts to express fibrosis-associated genes (Braga et al., 2015; Zhang et al., 2021). Once deposited, the ECM undergoes continuous remodeling by proteolytic enzymes secreted by macrophages, endothelial cells, and fibroblasts (Binnebösel et al., 2012). Studies have shown that inhibiting MMPs, key enzymes involved in ECM degradation, can reduce FBR and fibrosis in animal models (Jones et al., 2008; Witte et al., 1998; Figure 6; Table 3).

**TABLE 3:** Macrophage phenotype and function within the implant microenvironment during foreign body response.

Macrophage phenotype	M1 macrophage	M2 macrophage
Cell origin	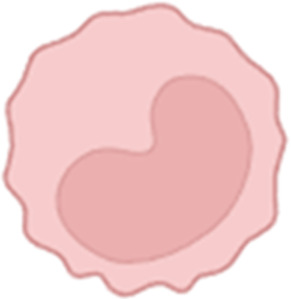 Monocyte	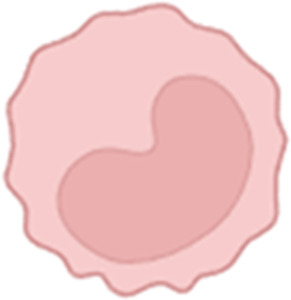 Monocyte
Inducer	IFNγ LPS	IL4 IL13
Functions	• Inflammatory response • Phagocytosis of fragments, dead cells • Angiogenesis initiation • Antigen presentation • Anti-fibrotic activity	• Activation of fibroblasts • ECM deposition • Fibrosis • Angiogenesis • Matrix remodeling
Result	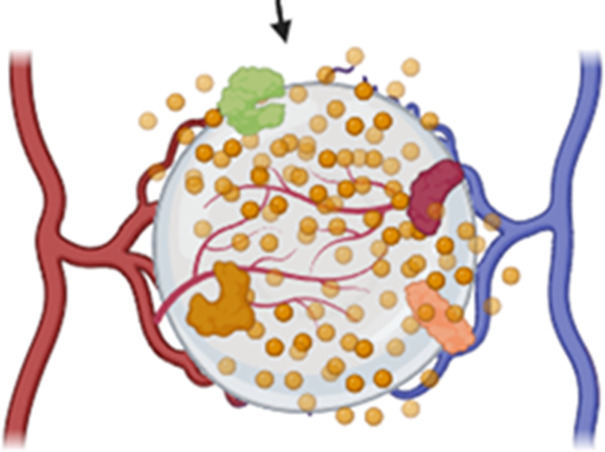 The inflammatory environment around the implant	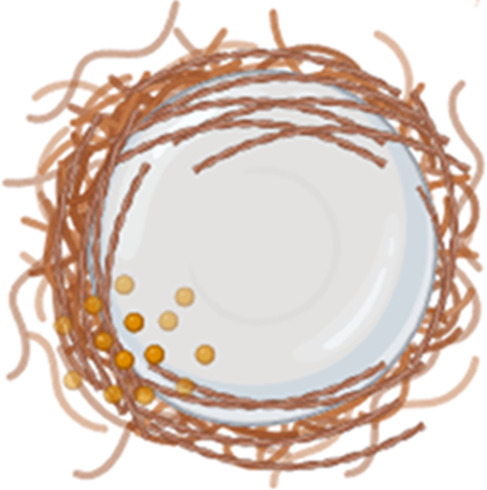 Fibrosis – formation of a thick capsule around the implant

Over time, the granulation tissue surrounding the implant matures into a thick collagenous capsule, composed initially of type III collagen and later replaced by type I collagen (Daneshgaran et al., 2023). This dense capsule effectively isolates the implant from the surrounding tissue, potentially hindering biointegration, and leading to implant failure. Despite extensive research, effectively preventing or reversing fibrous capsule formation remains a challenge. Systemic anti-inflammatory treatments have shown limited efficacy in eliminating capsule formation, and their potential side effects often outweigh their benefits (Klopfleisch and Jung, 2017; Klopfleisch, 2016; Witherel et al., 2019). Current strategies focus on local interventions, such as coating the implant surface with biomolecules or delivering anti-fibrotic drugs directly to the implant site (Piterina et al., 2009). While these approaches show promise in reducing capsule formation and inflammation, complete prevention remains elusive, and long-term studies are needed to assess their efficacy and safety (DiEgidio et al., 2014).

Alternative strategies, such as promoting the local accumulation of anti-inflammatory M2 macrophages, are also being explored as a potential means to modulate the FBR and reduce fibrosis without the drawbacks of systemic anti-inflammatory therapies (Ben-Mordechai et al., 2013). Ultimately, a deeper understanding of the complex interplay between implant surface properties, macrophage polarization, and the fibrotic process is crucial for developing effective strategies to promote implant biointegration and long-term success.

#### 4.5.1 Implant surface properties modulation of capsule formation

The extent of fibrous capsule formation around an implant is not uniform and is significantly influenced by the implant’s surface properties, particularly its topography and wettability (Kloss et al., 2011). These properties affect protein adsorption, cellular interactions, and the overall FBR, ultimately dictating the thickness and composition of the capsule.

Research by Glicksman et al. show that textured implants, particularly in the presence of ongoing shearing forces, can lead to formation of double capsules, a phenomenon associated with an increased risk of complications (Glicksman, 2021). Further research has revealed that variations in surface roughness and hydrophobicity can significantly impact implant biocompatibility and integration, potentially contributing to post-implantation complications (bin Anwar Fadzil et al., 2022; Souza et al., 2019; Munhoz et al., 2019; Barr et al., 2009).

The specific chemical composition of the implant surface also plays an important role. Hydrophilic surfaces containing both amino and hydroxyl groups have been linked to thicker capsule formation than other surface chemistries (Kamath et al., 2008; Tang et al., 1998). Conversely, the presence of carboxyl groups on hydrophobic surfaces has been associated with increased fibrosis and thicker capsules (Barbosa et al., 2006).

Strategies to minimize capsule formation often involve modifying the implant surface with anti-inflammatory materials (DiEgidio et al., 2014). For example, coatings incorporating hyaluronic acid (HA) or oxidized regenerated cellulose have been shown to attenuate capsule formation by modulating the inflammatory response and reducing fibroblast activity (Friedman et al., 2004; Lew et al., 2010).

Surface topography, particularly the presence and arrangement of pores, is another critical factor that influences capsule formation. Studies have shown that specific pore sizes and intranodal distances can promote thinner capsules and improve wound healing (Bota et al., 2010; Matlaga et al., 1976; Madden et al., 2010). For instance, implants with intranodal distances of 4.4 µm and pore sizes of 30–40 µm have been associated with reduced capsule thickness (Bota et al., 2010; Matlaga et al., 1976; Madden et al., 2010). Similarly, circular or ergonomically shaped implants with a surface roughness of approximately 4 µm have been shown to induce less fibrous capsule formation (Cui et al., 2006; Bota et al., 2010; Kanagaraja et al., 1996; Doloff et al., 2021).

In conclusion, the surface properties of an implant, including its topography, wettability, and chemical composition, exert a profound influence on capsule formation and the overall FBR. By optimizing these properties, it may be possible to modulate host response, minimize fibrosis, and promote successful implant integration and long-term clinical outcomes.

### 4.6 Summary and similarities between wound healing and the foreign body response: shared mechanisms in inflammation, remodeling, and fibrosis

The foreign body response is similar to the physiological process of wound healing, particularly in the initial phases, when both aim to restore tissue integrity and combat perceived threats (Babensee, 2020). While wound healing is an orchestrated repair mechanism following tissue injury, FBR represent an adaptive immune-mediated reaction to non-degradable foreign materials, often leading to encapsulation rather than full resolution (Anderson et al., 2008). Both processes exhibit overlapping stages, including acute inflammation, potential progression to chronic inflammation, ECM remodeling, and fibrosis, underscoring their evolutionary conservation as protective responses (Table 4).

**TABLE 4:** Structured comparison of stages of wound healing and foreign body response.

Wound healing stage	Shared characteristics	Foreign body response stage
Injury → Hemostasis (Clot formation)	Protein adsorption/Fibrin matrix	Biomaterial implantation → Protein adsorption
Inflammation → Proliferation (Granulation, Angiogenesis)	Acute Inflammation: Neutrophils/Macrophages, Cytokine release (TNF-α, IL-6)	Acute Inflammation: Neutrophils/Macrophages, Phagocytosis attempt
	Potential Chronic Inflammation: Persistent M1 macrophages, Sustained cytokines	Chronic Inflammation: Giant cells, Lymphocytes
	ECM Remodeling: MMPs/TIMPs, Collagen deposition	Granulation → ECM deposition
Remodeling (Collagen realignment)	Fibrosis: Excessive collagen, Scar/capsule	Fibrous encapsulation (Persistent fibrosis)

Wound healing occurs in four overlapping phases: hemostasis, inflammation, proliferation, and remodeling (Gonzalez et al., 2016). Hemostasis is initiated by platelet aggregation and fibrin clot formation, which provides a provisional matrix. In the inflammatory phase, neutrophils and macrophages are recruited to clear debris and pathogens, releasing cytokines, such as TNF-α and IL-6. The proliferation phase involves fibroblast activation, angiogenesis, and granulation tissue formation, with ECM deposition (primarily collagen III). Finally, remodeling replaces collagen III with collagen I and reorganizes the matrix for tensile strength, although excessive activity can result in hypertrophic scars. In contrast, FBR begins with protein adsorption on the biomaterial surface (seconds to minutes following implantation), followed by acute inflammation (lasting hours to days) akin to wound healing, where neutrophils and macrophages dominate and attempt phagocytosis (Chandorkar et al., 2018). If the material persists, this transition to chronic inflammation (lasting days to weeks) which is characterized by macrophage fusion into foreign-body giant cells (weeks to months) and lymphocyte infiltration. ECM remodeling is associated with fibroblast proliferation and collagen deposition, culminating in fibrosis via fibrous capsule formation (months to years) and isolation of the implant. In contrast to wound healing, FBR often lacks complete resolution and perpetuates low-grade inflammation.

The similarities between FBR and wound healing are also evident in their shared cellular and molecular characteristics (Babensee, 2020). In both processes, acute inflammation recruits innate immune cells for debris clearance, with macrophages polarizing from pro-inflammatory (M1) to anti-inflammatory (M2) phenotypes to facilitate repair. Chronic inflammation can arise if resolution fails due to infection in wounds or persistent biomaterials in FBR, which leads to sustained cytokine release and fibroblast activation. ECM remodeling involves MMPs and TIMPs, which balance degradation and synthesis; however, dysregulation promotes fibrosis in both, marked by excessive collagen accumulation and scar formation. For instance, TGF-β drives myofibroblast differentiation and ECM deposition in granulation tissue during wound proliferation and capsule formation during FBR. These parallels (Table 4) highlight opportunities for modulation; biomaterials mimicking native ECM can mitigate FBR by promoting M2 polarization and reducing fibrosis, akin to scarless fetal wound healing. However, divergences occur; wound healing typically resolves with functional tissue, whereas FBR chronicity can impair implant efficacy.

## 5 Silicone implants and complications

### 5.1 Silicone implant properties, foreign body response and clinical implications

Silicone breast implants, integral to aesthetic and reconstructive surgery, are categorized based on the average surface roughness: smooth (minimal roughness, Ra < 10 μm), macro-textured (aggressive roughness, Ra > 50 μm), and micro-textured (intermediate, Ra 10–50 μm, including nanotexture variants) (ISO 14607:2018) (ISO, 2018). These designs modulate the FBR, a host reaction involving inflammation, macrophage recruitment, and fibrous capsule formation, which can lead to complications such as capsular contracture (CC), and rarely, breast implant-associated anaplastic large cell lymphoma (BIA-ALCL). Smooth implants feature minimal roughness (Ra <10 μm) and appear irregular under microscopy with ripples approximately 5 μm wide. Smooth implants elicit a subdued FBR, forming thin, orderly capsules with aligned collagen fibers parallel to the surface (Capuani et al., 2022). Smooth implants often exhibit higher CC rates (6%–21% at 5–10 years) compared to textured (2%–10%) and micro-textured (1%–5%) implants, and this is attributed to denser, more aligned collagen deposition fostering contracture (Shin et al., 2018; Munhoz et al., 2019; Filiciani et al., 2022; Coleman et al., 1991). Comparative analyses reveal distinct patterns. For instance, a meta-analysis of over 16,000 patients reported CC rates of 6.8% for smooth versus 2.6% for textured implants (Filiciani et al., 2022; Coleman et al., 1991; Gorgy et al., 2023). In animal models, smooth surfaces yield thicker capsules (415 μm at 12 weeks) and denser collagen (67.8%) than textured variants with elevated myofibroblast infiltration (42.8%) and TGF-β1 expression, which are drivers of fibrosis (Jeon et al., 2022).

Textured (macrotextured) implants with Ra >50 μm and deeper pores (150–800 μm) were developed to disrupt collagen alignment and promote disorganized capsules to mitigate CC (Munhoz et al., 2019; Doloff et al., 2021). They enhance tissue integration via increased surface area (200–300 mm^2^) but intensify FBR through heightened macrophage activation and pro-inflammatory T-cell responses, leading to thicker scar tissue and chronic irritation (Munhoz et al., 2019; Doloff et al., 2021). This correlates with elevated biofilm formation (3-fold higher infection risk) and silicone particle release, which aggravates inflammation (Shin et al., 2018). *In vitro* and *in vivo*, macrotextures show moderate capsule thickness (261 μm at 12 weeks), but persistent fibrosis (Jeon et al., 2022). Microtextured implants (Ra 10–50 μm) balance these traits, with ∼1,800–2,200 contact points/cm^2^ and shallower depressions (40–100 μm). Microtextured implants suppress FBR more effectively, yielding thinner capsules (232 μm at 12 weeks), lower collagen density (46.2%), and reduced TGF-β1, minimizing inflammation while allowing guided integration (Munhoz et al., 2019; Jeon et al., 2022). Optimal roughness (∼4 μm) aligns with cellular scales, inhibiting pro-inflammatory pathways and scarring, as evidenced in rabbit and human studies (Doloff et al., 2021). A 30-patient study showed that low-micro (L-Micro) surfaces reduced myofibroblast activation and enhanced neovascularization compared to smooth (highest CC) or macro-textured (Huang et al., 2022).

Inflammation metrics further differentiate: smooth surfaces promote M2 macrophage polarization and anti-fibrotic IL-4, yet paradoxically higher CC due to shear forces and biofilm susceptibility; macro-textured surfaces increase M1-driven cytokines (TNF-α, IL-8), thickening capsules but disrupting alignment to lower contracture; micro-textured surfaces minimize both, with reduced FOXP3+ T-cell inhibition of fibrosis (Wells et al., 2024). A prospective study of 1,000 augmentations confirmed that the 1-year CC rate of smooth implants (4.5%) exceeded that of textured implants (1.8%), which is linked to bacterial adhesion differences (Filiciani et al., 2022). These outcomes bridge surface properties to clinical implications by elucidating how topography governs initial protein layers (e.g., fibronectin promotion on microtextures) and downstream FBR. Smooth surfaces facilitate sliding and dense fibrosis, heightening CC risk in dynamic tissues; textured disruption via anchorage, reducing migration but risking ALCL in macro variants; microtextured optimized integration, minimizing inflammation and contracture. This mechanistic insight informs design, for example, nano-engineering for hybrid surfaces, potentially halving CC incidence and enhancing safety and longevity.

The FBR’s intensity scales with roughness: smoother surfaces limit acute responses but risk contracture; rougher surfaces amplify chronic inflammation, potentially via bacterial synergy and immune dysregulation (Shin et al., 2018). This link underpins BIA-ALCL, a T-cell lymphoma associated with textured implants. In 2019, the FDA requested Allergan’s voluntary recall of Biocell macrotextured implants after linking them to 481 of 573 global BIA-ALCL cases and 33 deaths, citing a 6-fold higher risk than other textures (McKernan, 2021; Nelson et al., 2023). This prompted worldwide withdrawals, shifting clinical practice toward smooth and micro-textured alternatives. Post-2019, textured implant use plummeted with U.S. registries reporting less than 90% smooth/micro adoption in primary augmentations, with BIA-ALCL incidence stabilizing at approximately 1:30,000 for remaining textures (Swanson, 2023).

### 5.2 Implant failure: silicone implants versus others

Silicone breast implants exhibit significant failure rates over time, primarily driven by the host FBR. Implant failure includes rupture, leakage, and capsular contracture, with a cumulative incidence escalating with implant duration. Longitudinal studies indicate rupture rates of 6%–24% at 10 years post-implantation (Hillard et al., 2017). For instance, a prospective MRI cohort reported a 6.4% rupture rate for primary augmentation and 5.2% for revision augmentation at 8 years (Hillard et al., 2017). Another analysis of MemoryGel implants showed a 24% Kaplan-Meier estimated rupture rate at 10 years (Paolini et al., 2023). Globally, older implants demonstrate higher failure rates: 30% at 5 years, 50% at 10 years, and 70% at 17 years, with an annual rate of approximately 6% in the first 5 years (Marotta et al., 1999). A retrospective study estimated a 15.1% incidence of rupture, with a mean implant lifespan of 10.1 years (Paolini et al., 2023). These statistics underscore the time-dependent degradation, which is exacerbated by mechanical stress and material fatigue.

In rupture scenarios, silicone gel extrudes, eliciting intensified macrophage-driven FBR, including granuloma formation and systemic silicone migration. Gel bleed-microscopic silicone diffusion through intact shells further sustains low-grade inflammation, accelerating capsular contracture (Baker grades III-IV) in up to 50% of cases by 10 years (Moyer et al., 2012). Quantitative proteomics revealed that acute wound responses evolve into persistent fibrosis, contributing to device failure (Schoberleitner et al., 2023). Thus, FBR not only precipitates mechanical breach, but also biomechanical distortion, necessitating intervention.

Revision surgery due to implant failure is common, with U.S. FDA data indicate that 20% of women require removal within 10 years, rising to approximately 50% over 15 years, often for rupture or contracture (Baek et al., 2014). A prospective U.S. cohort reported revision rates of 1.6% for cosmetic implants and 11.8% for reconstructive implants, although these underrepresented long-term failures (Lieffering et al., 2022). Globally, explantation for objective failure (e.g., rupture) accounts for 34% of primary augmentation and 47.6% of revisions, with 14 estimated removal risk of 14% at 8 years (Zhang et al., 2023). In Europe, PIP implant studies showed 21.3% ruptures per implant, leading to 35.2% patient revisions (Quaba and Quaba, 2013). These figures highlight the substantial burden, emphasizing the need for advanced biomaterials to mitigate FBR and reduce revision rates.

Silicone breast implant failure, primarily rupture or capsular contracture, exhibits a higher prevalence than other implants, with U.S. and global rates showing 7.8% cumulative rupture at 10 years for primary augmentation. In contrast, orthopedic implants, such as hip replacements, demonstrate lower failure rates. U.S. and global survivorship reaches 90%–95% at 10 years, with revision rates of <5% at 10 years and 4.56% failure overall (Kenney et al., 2019; Springer et al., 2009). Metal-on-metal variants show 6.2% failure at 5 years globally, but modern designs last 15–20 years with approximately 10% failure (Park et al., 2018; Ebramzadeh et al., 2011). Knee replacements mirror this, with U.S. revision rates of 5.66% at 5 years and 96.1% survivorship at 10 years globally, although poor outcomes affect 7%–20% due to infection or loosening (Deere et al., 2021).

Dental implants have markedly lower failure, at 2%–4% globally and in the U.S., with 96%–97% survivorship at 10 years; peri-implantitis drives most cases (3.1%) (Iacono et al., 2022; Lombardo et al., 2023). Cardiac pacemakers exhibit the lowest device malfunction, 0.16%–0.6% in U.S. recalls, although complications such as lead issues necessitate reoperation in 7.9% of cases globally (El-Chami, 2021).

Overall, silicone breast implants fail more frequently (5%–50% over 10–15 years) than orthopedic (5%–12% at 10 years), dental (2%–5%), or cardiac (<1%) implants, highlighting softer tissue dynamics and immune responses as key differentiators in the U.S. and global contexts.

## 6 Targeting implant-induced foreign body responses

Mitigating the FBR and its associated complications, such as fibrous encapsulation and capsular contracture, is a critical goal in optimizing the long-term success of implantable medical devices (Carnicer-Lombarte et al., 2021; Capuani et al., 2022). Various strategies are under investigation, targeting different stages of FBR, from the initial protein adsorption to the chronic inflammatory and fibrotic phases. These strategies can be broadly categorized into: 1 modification of the implant surface, 2 modulation of the systemic immune response, and 3 control of the local immune response at the implant site (Carnicer-Lombarte et al., 2021; Capuani et al., 2022).

### 6.1 Modification of the implant surface

This approach aims to engineer an implant surface to minimize protein adsorption, reduce immune cell adhesion, and promote tissue integration.

Strategies include:

*Physical Modification:* Altering surface roughness, topography, and porosity influences protein adsorption and cellular interactions.

*Chemical Modification:* Utilizing surface coatings with specific chemical properties to modulate protein adsorption, cell adhesion, and inflammatory responses.

*Biomimetic Modification:* Emulating the natural ECM to promote tissue integration and reduce the perception of the implant as a foreign object.

### 6.2 Modulation of the systemic immune response

This approach aims to dampen the overall immune reaction to the implant, reducing inflammation and fibrosis. Strategies include:

*Immunosuppressive Drugs:* Utilizing systemic immunosuppressants to reduce the overall immune response, although this approach can have significant side effects and may impair wound healing.

*Immune Tolerance Induction:* Developing strategies to induce specific immune tolerance to the implant material and minimizing the FBR without compromising overall immune function.

### 6.3 Control of the local immune response

This approach focuses on modulating the immune response specifically at the implant site, minimizing inflammation and fibrosis, while preserving overall immune function. Strategies include:

*Local Drug Delivery:* Delivering anti-inflammatory or anti-fibrotic drugs directly to the implant site, minimizing systemic side effects.

*Decellularized ECM:* Utilizing decellularized ECM materials to modulate the local immune response, promoting constructive remodeling and tissue regeneration.

*Cell-Based Therapies:* Seeding the implant with specific cell types, such as regulatory T cells or mesenchymal stem cells, to actively modulate the immune response and promote tissue regeneration.

Importantly, the fibrotic response to implants is similar to organ fibrosis, suggesting that anti-fibrotic therapies, such as pirfenidone and pan-v integrin inhibitors, may be promising in preventing capsular contracture and preserving implant function (Gancedo et al., 2008; Love and Jones, 2009). A multifaceted approach targeting different stages of the FBR is likely required to achieve optimal implant biocompatibility and long-term success. Ongoing research is focused on developing and refining these strategies, with the ultimate goal of creating implants that seamlessly integrate with the host tissue, minimizing complications and maximizing patient benefit.

### 6.4 Biomimetic coating/modification of implants to modulate the foreign body response

While traditional approaches to mitigate fibrosis and scar tissue formation often rely on immunosuppressive medications with potential adverse effects (Marcolongo et al., 2004; Doloff et al., 2017), biomimetic strategies offer a promising alternative (Taraballi et al., 2018; Noskovicova et al., 2021b). By emulating the natural ECM, these strategies aim to render implants less “visible” to the immune system, promoting tissue integration and reducing the FBR.

The ECM is a network of proteins and polysaccharides that provide support and biochemical cues that regulate cellular behavior. Biomimetic approaches leverage this knowledge by modifying implant surfaces with ECM-derived components to create a more biocompatible interface (Li et al., 2021; Schulz et al., 2014). These modifications can influence all phases of the FBR, from initial protein adsorption to chronic inflammation and fibrous encapsulation.

*ECM Protein Coatings:* Coating implant surfaces with specific ECM proteins can modulate cellular interactions and promote tissue integration (Li et al., 2021; Schulz et al., 2014). Fibronectin, a ubiquitous ECM glycoprotein, plays a crucial role in cell adhesion, migration, and differentiation (Cowles et al., 1998; Moursi et al., 1997; Pankov and Yamada, 2002). It exists in both soluble and insoluble forms, with the soluble form found in plasma and the insoluble form associated with cells and the ECM (Pankov and Yamada, 2002). Research has shown that fibronectin, along with other cell-binding proteins like collagen and laminin, can enhance the differentiation of various cell types *in vitro* (Padhi and Nain, 2020; Czyz and Wobus, 2001).

Various strategies have been employed to enhance fibronectin deposition on implant surfaces, including direct adsorption, covalent immobilization, and the use of protein-binding substrate layers (Aota et al., 1994; Cutler and García, 2003; Lin et al., 2015; Ghadhab et al., 2021). For instance, fibronectin can be directly deposited or adsorbed onto the implant surface, but this approach may be limited by the potential for protein desorption over time (Aota et al., 1994; Cutler and García, 2003; Lin et al., 2015). To address this, a protein-binding substrate layer, such as polydopamine, can be applied to the implant surface before adding fibronectin, enhancing its retention and stability (Cutler and García, 2003; Ghadhab et al., 2021).

Another approach involves utilizing recombinant protein fragments or short peptides containing specific cell-binding motifs, such as the RGD or LDV sequences found in fibronectin (Martino et al., 2011; Petrie et al., 2009; García et al., 2002; Benoit and Anseth, 2005; Leahy et al., 1996). These motifs interact with integrin receptors on cell surfaces, promoting cell adhesion and spreading. An animal study demonstrated that coating implants with fibronectin in combination with IL-4 resulted in thinner capsules, likely due to the promotion of M2 macrophage polarization (Tan et al., 2020). This highlights the potential for combining ECM protein coatings with immunomodulatory factors to further enhance implant biocompatibility.

*Glycosaminoglycan (GAG) Coatings:* GAGs, another major component of the ECM, also hold potential for modulating the FBR. Hyaluronic acid and heparin, for example, have demonstrated anti-inflammatory properties by activating regulatory T cells and suppressing macrophage activation (Juhas et al., 2015; Tian et al., 2017). Coating amino-terminated silicone with these GAGs resulted in downregulation of the NF-κB signaling pathway, an important pathway in the regulation of inflammation (Juhas et al., 2015; Tian et al., 2017).

Gelatin, a denatured form of collagen, has also been explored as a coating material. While gelatin coatings alone may lack long-term stability (Burugapalli et al., 2018), combining gelatin with hyaluronic acid can improve mechanical properties and reduce fibrotic tissue formation (Joo et al., 2021). This combination leverages the biocompatibility of gelatin and the anti-inflammatory properties of hyaluronic acid. Heparin coatings on artificial vascular grafts have been shown to enhance angiogenesis and promote M2 macrophage polarization (Kim et al., 2019).

*Aptamers:* Aptamers, small oligonucleotides that bind to specific target molecules with high affinity, offer an intriguing alternative to monoclonal antibodies for surface modification (Boshtam et al., 2017). They possess several advantages, including low immunogenicity, low toxicity, and cost-effective production (Radom et al., 2013). Studies have investigated the use of ssDNA aptamers against ECM proteins, including fibronectin, in hydrogels to enhance cell adhesion (Galli et al., 2016; Parisi et al., 2017; Parisi et al., 2019; Abune et al., 2022). These aptamers bind strongly to fibronectin, promoting cell attachment and potentially modulating the FBR.

Biomimetic strategies utilizing ECM-derived components, such as proteins, GAGs, and aptamers, hold significant promise for improving implant biocompatibility and reducing the FBR. By mimicking the natural cellular microenvironment, these approaches aim to promote tissue integration, modulate the immune response, and ultimately enhance the long-term success of implantable medical devices.

### 6.5 Modulation of the foreign body response with decellularized ECM

Decellularized ECM have emerged as a promising tool for modulating the FBR and promoting implant biocompatibility. While historically used as scaffolds for tissue reconstruction, cell delivery, and controlled release of therapeutic molecules (Wolf et al., 2015), their role in actively modulating the immune response is gaining increasing recognition (Mariani et al., 2019; Liang et al., 2023). Preclinical studies have highlighted the immunomodulatory potential of decellularized ECM scaffolds, although the underlying mechanisms are still being elucidated (Badylak et al., 2016; Yu et al., 2016).

The decellularization process effectively removes cells and immunogenic components from the native tissue while preserving the intricate architecture and biochemical composition of the ECM (Dzobo et al., 2019; Badylak et al., 2009; Turner and Badylak, 2015). This creates a biocompatible scaffold that can support tissue regeneration and modulate the host immune response (Turner and Badylak, 2015). The presence of decellularized ECM at the implant site can promote constructive remodeling, influence the behavior of infiltrating immune cells like neutrophils and macrophages, and ultimately guide the FBR towards a more regenerative outcome (Hong et al., 2020; Qiu et al., 2018).

One of the key mechanisms by which decellularized ECM modulates the FBR is through its influence on macrophage polarization. Studies have shown that decellularized ECM can shift macrophage phenotypes towards an M2-like profile, characterized by reduced inflammation and enhanced tissue repair (Hong et al., 2020; Qiu et al., 2018). This M2 polarization is associated with less scarring and greater constructive remodeling compared to cellular scaffolds (Brown et al., 2012). Furthermore, decellularized ECM can create a Th2-dominant immune microenvironment, which further promotes M2 macrophage polarization via an IL-4-dependent pathway (Sadtler et al., 2016). This suggests that inducing a Th2 response is a key aspect of the immunomodulatory effects of decellularized ECM.

It is important to note that the specific decellularization method used can influence the immunomodulatory properties of the resulting ECM. Macrophages can recognize and respond to denatured or damaged collagen, highlighting the importance of optimizing decellularization protocols to preserve the native ECM structure and ensure a favorable immune response (Gowen et al., 2000; Veres et al., 2015). Moreover, the source of the decellularized ECM can also influence its effects on macrophages, with different tissue sources eliciting varying responses (Dziki et al., 2017; Keane et al., 2017). For instance, several studies have demonstrated that decellularized ECM from different tissues can induce an M2 macrophage phenotype similar to that observed with IL-4 stimulation (Witherel et al., 2021; O'Brien and Spiller, 2022; Sicari et al., 2014).

The incorporation of decellularized ECM or its derived components into implant design has shown promise in promoting implant tolerance and reducing the severity of the inflammatory response (Badylak et al., 2009). This ability to regulate inflammation through macrophage polarization is a major focus of research exploring the use of decellularized ECM in implantable medical devices (Liang et al., 2023; Aamodt and Grainger, 2016; Dong et al., 2021). Studies have shown that decellularized ECM can improve healing responses, characterized by reduced M1 macrophage infiltration and increased M2 polarization, as confirmed by immunohistological evaluations (Brown et al., 2009; Fishman et al., 2013).

Beyond its immunomodulatory effects, decellularized ECM can also serve as an effective delivery vehicle for therapeutic molecules and drugs, further enhancing its potential for promoting tissue regeneration and modulating the FBR (Taylor et al., 2018; Zhang et al., 2022; Saleh et al., 2018). In conclusion, decellularized ECM represents a versatile and promising biomaterial for modulating the FBR and promoting implant biocompatibility. Its ability to support tissue regeneration, modulate macrophage polarization, and serve as a drug delivery vehicle highlights its potential for improving the long-term success of implantable medical devices.

## 7 Targeting implant-associated fibrosis

Addressing fibrosis and preventing its progression is critical for successful implant integration and long-term functionality. Strategies aimed at suppressing myofibroblast activity or preventing their activation are crucial for counteracting the excessive deposition of ECM that characterizes fibrosis (Lodyga and Hinz, 2020; Daskalopoulos et al., 2013).

Several factors contribute to fibrosis in the context of the FBR. Aberrant M2 macrophage activity, for instance, can promote a profibrotic environment by releasing factors that stimulate fibroblast activation and ECM production (Major et al., 2015; Noskovicova et al., 2021a; Kzhyshkowska et al., 2015; Mariani et al., 2019; Tschumperlin and Lagares, 2020). Additionally, fibroblasts possess the ability to sense and respond to mechanical cues from their environment, including the stiffness of the implant material (Major et al., 2015; Noskovicova et al., 2021a; Kzhyshkowska et al., 2015; Mariani et al., 2019; Tschumperlin and Lagares, 2020). This mechanosensing can trigger fibroblast activation and differentiation into myofibroblasts, further contributing to fibrosis.

Targeting specific molecular pathways involved in myofibroblast activation and fibrosis offers promising therapeutic avenues. Integrins, transmembrane receptors that mediate cell adhesion and signaling, are prime targets for anti-fibrotic therapies (Hintermann and Christen, 2019; Schnittert et al., 2018). Specific integrins, particularly those containing the β subunit, play a crucial role in activating latent TGF-β1, a potent profibrotic factor that drives myofibroblast differentiation (Annes et al., 2002). Inhibiting these integrins or blocking TGF-β1 signaling can effectively reduce fibrosis.

Another key pathway involved in myofibroblast activation and contraction is the Rho/ROCK signaling cascade (Martinac, 2014). This pathway regulates actin-myosin contractility, a key driver of myofibroblast-mediated tissue contraction and fibrosis. Inhibiting Rho/ROCK signaling can disrupt myofibroblast function and attenuate fibrosis. The Hippo signaling pathway also play a significant role in myofibroblast activation and fibrosis (Dasgupta and McCollum, 2019; Rausch and Hansen, 2020). YAP and TAZ are transcriptional coactivators that promote the expression of profibrotic genes. Inhibiting upstream regulators of YAP/TAZ signaling, such as specific G protein-coupled receptors (GPCRs), has shown promise in blocking myofibroblast activation and fibrosis (Haak et al., 2019).

Targeting these various mechanotransduction and signaling pathways involved in myofibroblast activation holds great potential for alleviating peri-implant fibrosis. Given that implants are often perceived as stiff by surrounding cells, disrupting these mechanosensitive pathways may be crucial for promoting implant integration and long-term success.

Fibrosis is a complex process driven by a multitude of factors, including macrophage activity, fibroblast mechanosensing, and various signaling pathways. Targeting these pathways with specific inhibitors or modulators offers promising therapeutic strategies for reducing fibrosis, improving implant biocompatibility, and preventing complications such as capsular contracture.

### 7.1 Antifibrotic drugs

Pharmacological interventions targeting specific mediators and pathways involved in fibrosis offer promising strategies for mitigating capsule formation and improving implant outcomes. Several anti-fibrotic drugs have been investigated for their potential to modulate the FBR and reduce excessive scar tissue formation.

*Glucocorticoids*: Glucocorticoids, a class of steroid hormones, are potent anti-inflammatory agents that exert their effects by suppressing the expression of pro-inflammatory cytokines, such as TNF-α and IL-1β (Schleimer, 1993; Joyce et al., 1997). They also inhibit the expression of molecules involved in leukocyte chemotaxis and adhesion, reducing the infiltration of immune cells to the implant site (Cain and Cidlowski, 2017). By suppressing the inflammatory response, glucocorticoids indirectly reduce fibroblast recruitment and activation, thereby limiting fibrosis (Jeon et al., 2018; Kastellorizios et al., 2015). However, their use is limited by potential side effects, including muscle wasting and immunosuppression, particularly with long-term administration (Oray et al., 2016). Triamcinolone, another steroid with anti-fibrotic properties, is also used to control implant-associated fibrosis, but its continuous use is not recommended due to potential adverse effects.

*Tranilast*: Tranilast, an anti-allergic drug commonly used to treat asthma and hypertrophic scarring, has shown promise in reducing implant-associated fibrosis. It acts by inhibiting TGF-β secretion and its downstream signaling cascade, effectively suppressing collagen synthesis and fibroblast activation (Takahashi et al., 2018; Miyazawa et al., 1995). Studies have demonstrated that tranilast can reduce capsule formation around silicone implants, particularly when administered early after implantation (Park et al., 2015).

*Leukotriene Receptor Antagonists*: Cysteinyl leukotrienes, lipid mediators involved in inflammation, play a role in fibroblast recruitment and differentiation into myofibroblasts during the FBR (Singh et al., 2010; Guimaraes et al., 2018; Kanaoka and Boyce, 2004; Wahl, 1992). Montelukast and zafirlukast, leukotriene receptor antagonists commonly used to treat asthma, have demonstrated anti-fibrotic effects by blocking leukotriene signaling (Diamant et al., 1999; Zhou et al., 2019). Montelukast, in particular, binds to the CysLT1 receptor on polymorphonuclear cells and has been shown to reduce fibroblast and myofibroblast numbers and inhibit collagen production (Peng et al., 2017; Dong et al., 2023). Both montelukast and zafirlukast have been shown to prevent capsule formation after silicone breast implantation in animal models and clinical studies (Dong et al., 2023; Moreira et al., 2009; Kang et al., 2015; Peters-Golden and Henderson, 2007; Muraki et al., 2009; Kim et al., 2017; Altinbas et al., 2015; Spano et al., 2008; Bastos et al., 2007).

*Halofuginone*: Halofuginone, another anti-fibrotic compound, interferes with Smad3 phosphorylation, a key step in the TGF-β signaling pathway, thereby inhibiting collagen synthesis and fibroblast activation (Pines and Nagler, 1998; Granot et al., 1993). While halofuginone has shown efficacy in reducing collagen levels and capsule thickness around implants, its systemic use is limited due to potential side effects (Zeplin et al., 2010; Jordan and Zeplin, 2012; Olbrich et al., 2005). Local delivery of halofuginone to the implant site may offer a more targeted approach with reduced systemic toxicity.

In summary, various anti-fibrotic drugs targeting different mediators and pathways involved in fibrosis have shown promise in preclinical and clinical studies. While challenges remain in terms of efficacy, safety, and optimal delivery methods, these pharmacological interventions offer valuable tools for modulating the FBR, reducing capsule formation, and improving the long-term success of implantable medical devices.

## 8 Summary

The FBR is an unavoidable consequence of introducing any foreign material, including silicone breast implants, into the human body. This complex biological process, while sharing similarities with wound healing, ultimately aims to isolate the implant from the surrounding tissues by encapsulating it within a fibrous capsule. While this response is intended to be protective, it can lead to adverse outcomes, such as chronic inflammation, fibrosis, implant failure, and even rejection. Therefore, a deeper understanding of the cellular and molecular mechanisms driving the FBR is crucial for improving implant biocompatibility and long-term clinical success. The FBR is a dynamic and multifaceted process involving a complex interplay of cell types, signaling molecules, and the ECM. The initial interaction between the implant surface and host proteins is critical, as the adsorbed protein layer acts as a “molecular fingerprint” that influences subsequent cellular interactions. Immune cells, particularly macrophages, play a central role in orchestrating the FBR, exhibiting remarkable plasticity in their polarization into different phenotypes with distinct functions. The balance between pro-inflammatory and pro-healing macrophage phenotypes significantly influences the trajectory of the FBR and the extent of fibrosis.

Implant surface properties, including topography, chemical composition, and mechanical properties, play a crucial role in modulating the FBR. These properties affect protein adsorption, cellular adhesion, activation, and differentiation, ultimately influencing the overall tissue response. Other factors, such as implant design, surgical technique, and mechanical loading, also contribute to the complex interplay of events that determine the fate of an implant.

While our understanding of the FBR has significantly advanced, there are still critical knowledge gaps. We are currently unable to fully orchestrate the individual processes involved in the FBR to create an optimal environment for implant biointegration and achieve ideal host responses. This highlights the need for continued research to unravel the intricate mechanisms underlying the FBR and develop strategies to effectively modulate this response.

## 9 Future outlook

Future research should focus on several key areas to advance our understanding and management of the FBR.

### 9.1 Deciphering the complex interplay of cellular and molecular events

A more comprehensive understanding of the intricate signaling pathways, cellular interactions, and dynamic changes in the ECM during the FBR is needed. This includes further investigation of macrophage polarization, the role of other immune cells, and the interplay between inflammation and fibrosis.

### 9.2 Optimizing implant surface properties

Developing novel biomaterials and surface modification strategies to minimize protein adsorption, reduce immune cell activation, and promote tissue integration is crucial. This includes exploring biomimetic approaches that emulate the natural ECM and utilizing advanced surface characterization techniques to understand the impact of surface properties on the FBR.

### 9.3 Developing targeted therapies

Identifying and targeting specific molecular pathways involved in inflammation and fibrosis can lead to more effective and less invasive treatments for FBR-related complications. This includes exploring novel anti-inflammatory and anti-fibrotic drugs, as well as cell-based therapies that can actively modulate the immune response.

### 9.4 Personalized medicine approaches

Investigating the role of individual patient factors, such as genetics and immune status, in the FBR can pave the way for personalized implant strategies and therapies tailored to individual needs.

### 9.5 Advanced *In vitro* and *In vivo* models

Developing more sophisticated *in vitro* and *in vivo* models that accurately recapitulate the complex dynamics of the FBR is essential for testing novel biomaterials and therapeutic interventions. This includes utilizing 3D tissue models, organ-on-a-chip platforms, and humanized animal models.

## 10 Conclusion

The FBR is a complex and unavoidable consequence of implant placement, posing significant challenges to achieving optimal implant integration and long-term success. While our understanding of the FBR has grown considerably, there is still much to learn about the intricate interplay of cellular and molecular events that govern this response. Future research focused on deciphering these mechanisms, optimizing implant surface properties, developing targeted therapies, and utilizing personalized medicine approaches holds the key to improving implant biocompatibility and transforming the future of implantable medical devices.

## Glossary

3D: three-dimensional
ALCL: anaplastic large cell lymphoma
αMβ2: integrin alpha M beta-2
αVβ1: integrin alpha V beta-1
CCL2: chemokine C-C motif ligand 2
CD: cluster of differentiation
DAMPs: damage-associated molecular patterns
E: elastic modulus
ECM: extracellular matrix
FBR: foreign body response
FBGCS: foreign body giant cells
FDA: Food and Drug Administration
HA: hyaluronic acid
IgG: immunoglobulin G
IL: Interleukin
ISO: International Organization for Standardization
MCP-1: monocyte chemoattractant protein-1
MMP: matrix metallopeptidase
NET: neutrophil extracellular traps
PDGF: platelet-derived growth factor
PDMS: polydimethylsiloxane
PEG: poly (ethylene glycol)
PGA: poly-glycolic acid
PLA: poly-lactic acid
PLGA: poly-lactic-co-glycolic acid
PLL: poly-l-lysine
PMAA: poly (methacrylic acid)
PMNs: polymorphonuclear leukocytes
qPCR: quantitative polymerase chain reaction
Ra: arithmetic mean height
RGD: arginine–glycine–asparagine
TGF-β: transforming growth factor beta
TIMP4: metalloproteinase inhibitor 4
TNF-α: tumour necrosis factor α
WHO: World Health Organization
